# Integrative Omics Analysis Reveals the Regulation of Hypoxia Tolerance in Large Yellow Croaker (*Larimichthys crocea*) via the Lipoic Acid Synthase (*lias*) Gene

**DOI:** 10.1002/advs.76310

**Published:** 2026-06-26

**Authors:** Jie Ding, Songpeng Jia, Ran Meng, Xuelei Wang, Xiongfei Wu, Weiliang Shen, Yibo Zhang, Junquan Zhu

**Affiliations:** ^1^ Key Laboratory of Marine Biotechnology of Zhejiang Province Key Laboratory of Aquacultural Biotechnology Ministry of Education College of Marine Sciences Ningbo University Ningbo Zhejiang China; ^2^ Zhejiang Key Laboratory of Aquatic Germplasm Resources Ningbo Academy of Oceanology and Fishery Ningbo Zhejiang China

**Keywords:** DNA methylation, GWAS, HIF‐1α, hypoxia, large yellow croaker, lipoic acid synthase

## Abstract

Hypoxia stress seriously affects the survival of aquatic animals. However, little is known about the regulation mechanism under hypoxia stress; hence, it is important to fully clarify this regulation to improve the hypoxia tolerance of aquatic animals. In this study, we integrated multi‐omics data from *Larimichthys crocea* under hypoxic stress and validated the functions of candidate genes using multiple techniques. Genome‐wide association study and transcriptome analysis identified lipoic acid synthase (*lias*) as an important candidate gene for hypoxia tolerance. Specifically, SNP in the second intron region of *lias* significantly altered the transcription levels between major haplotypes. Methylation and CUT&Tag sequencing confirmed that this significant SNP could alter the enhancer activity of *lias* by affecting the methylation level, and eventually affecting *lias* expression. Functional characterization revealed that *lias* can inhibit the production of α‐ketoglutaric acid under hypoxia, and then negatively regulate the protein level of hypoxia‐inducible factor (HIF)‐1α through prolyl hydroxylase domain, resulting in less hydroxylation dependent degradation of HIF‐1⍺ and more downstream target gene expression of Hap2, leading to better adaptation to the hypoxia environment. Our study provides a new research perspective for the theory of fish hypoxia tolerance.

## Graphical abstract

Lipoic acid synthase (*lias*) can regulate α‐KG levels through lipoylation, thereby negatively regulating HIF‐1α protein levels via PHD under hypoixa. The Hap2 allele of lias exhibits lower expression levels than Hap1, leading to the accumulation of more HIF‐1α protein and thereby enhancing hypoxia tolerance.

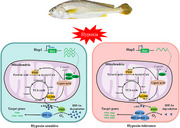

## 1 Introduction

Dissolved oxygen (DO) is a key environmental factor needed for the survival of various aquatic animals. However, since oxygen is poorly soluble in water, the DO content in natural water is only 1/34th of that in air. Moreover, the DO content in water is drastically influenced by various factors, such as temperature variations, eutrophication of the water body, and environmental pollution, which exposes aquatic animals to the adverse effects of hypoxia during their growth, development and reproduction [1]. Hypoxia is defined as a DO content of less than 2 mg/L in water [2]. Recently, hypoxia has become a major challenge for global aquatic ecosystems. More than 500 hypoxia zones, covering thousands of square kilometers, have appeared in global oceans, and some offshore areas have even become permanently hypoxic. Climate change can potentially exacerbate the problem, with inevitable consequences for aquaculture development [3].

The large yellow croaker (*Larimichthys crocea*) is a seawater fish endemic to the southeastern coast of China, and is named after the “cackling” sound it makes during the reproductive season [4]. *L. crocea* is consumer‐friendly, owing to its delicious taste, rich nutrition, and attractive golden body color; it has a very high market value and is considered as the “national fish”. Recently, with the continuous development of aquaculture, *L. crocea* has become the major sea‐cage farmed fish in China, exhibiting the highest national yields among seawater aquaculture fish [5]. However, due to factors such as high‐density sea‐cage farming, water pollution, and red tides, the DO concentration in the cages often falls below 3 mg/L, and during summer, it can even fall below 2 mg/L (Figure S1). This leads to slow growth, decreased immunity, and even death of *L. crocea*, causing serious issues for the fish farming industry. Therefore, comprehensive analysis of the response and tolerance mechanism of *L. crocea* to hypoxia stress will not only provide new research perspectives for hypoxia tolerance in fish [6], but also identify precise targets for developing hypoxia‐tolerant molecular markers and gene editing breeding [7], which are of great theoretical significance and potential application value.

Currently, few studies have investigated hypoxia in *L. crocea*, to determine the genetic basis and molecular mechanisms of hypoxia tolerance. For example, transcriptomic sequencing of the brain under hypoxia in *L. crocea* revealed that the hypothalamic‐pituitary‐adrenal axis‐related genes play a crucial role in reducing inflammatory responses and inhibiting energy metabolism [8]. Long non‐coding RNA (lncRNA) sequencing of the liver has identified an lncRNA (Linc_06633.1) in the upstream antisense region of prolyl hydroxylase domain 2 (*phd2*), which may serve as an important regulatory factor promoting *phd2* expression under hypoxia [9]. Methylation analysis of the gills has highlighted that hypoxia‐inducible factor (HIF)‐1 pathway‐related genes (*hif‐1α*, *hif‐3α*) play important roles in hypoxia tolerance [10]. Further, proteomics analysis has revealed that proteins associated with the extracellular matrix‐receptor interaction, along with protein digestion and absorption pathways are significantly enriched under hypoxia [11]. Gene family analysis has revealed that the copper metabolism gene MURR1 domain gene family exhibits significant changes under hypoxia [12]. Additionally, genome‐wide association study (GWAS) in *L. crocea* revealed that erythropoiesis‐associated genes, such as *ho1*, *klf3*, *thsd4*, and *melk*, are closely related to hypoxia tolerance in *L. crocea* [5]. These studies provide key genetic clues for understanding the hypoxia tolerance of *L. crocea*, however, the single‐technique approach limits the research perspective to a specific molecular level. This makes it impossible to distinguish whether transcriptional regulation, post‐transcriptional mechanisms, or metabolic adaptations play the dominant role, thereby making it difficult to identify the key molecules directly responsible for physiological functions. Furthermore, due to sample size limitations, the study was unable to effectively link genetic variations (particularly low‐frequency mutations) to hypoxic tolerance phenotypes, leaving the underlying key mutations and their regulatory networks unclear.

This study aims to explore key candidate genes and genetic mechanisms controlling the hypoxia tolerance phenotype of *L. crocea*. First, single nucleotide polymorphism (SNP) chip data from different *L. crocea* populations, whole genome sequencing (WGS) analysis, and transcriptome analysis data were integrated to identify key genetic variations and genes associated with hypoxia tolerance in *L. crocea*. Subsequently, methylation and histone modification analysis were used to investigate how these genetic variations affect gene expression. Finally, gene function validation was performed using interference and overexpression experiments in *L. crocea* cell lines. The study findings are expected to provide novel insights into the genetics of breeding new hypoxia‐tolerant varieties of *L. crocea* and promote innovation in aquaculture technology [13].

## 2 Materials and Methods

### 2.1 Ethics Statement

All the procedures involving *L. crocea* used in this study were in strict accordance with the guidelines for experimental animals formulated by Ningbo University and were approved by the Animal Care and Use Committee of Ningbo University (reference number NO.14390).

### 2.2 Sample Collection

From 2020 to 2023, a total of 4,347 *L. crocea* samples (body weight 49.47 ± 8.47 g, body length 14.12 ± 4.19 cm) from four populations were included in the study. Among these, 1813 samples from the DaiQu group (DQ_1) and 1514 samples from the MinDong group (MD_1) were bred naturally, whereas 514 samples from the DaiQu group (DQ_2) and 506 samples from the MinDong group (MD_2) were subjected to hypoxia‐tolerant selective breeding (Table S1). These *L. crocea* were obtained from the sea cage in the Xiangshan Bay (29° 29' N and 121° 34' E), Ningbo, Zhejiang Province, China, and their breeding methods and culture environment (including water source, temperature, salinity, pH, DO, feed types, feeding frequency and cycle, and light cycle) were consistent. They were then transported to an indoor circular tank for 14 d. The fish were acclimatized in the circular tank (r = 1.2 m, h = 1.1 m) for 12 h before the start of the experiment. Initially, the tank was covered with a transparent plastic film to isolate the effect of oxygen from the air, and then the gradient reduction method previously employed in the laboratory was adopted to reduce the DO [14]. DO was reduced from normoxia (approximately 7–8 mg/L) to 6 mg/L within 1 h using nitrogen and maintained for 2 h; it was further reduced to 2 mg/L at a rate of 0.5 mg/L every 2 h, and then consistently reduced at a rate of 0.1 mg/L every 2 h until the DO content was zero or the fish died completely. An in‐house automated DO controller was used to maintain the stability of DO in the water during the experimental period (Figure S2). Two mini submersible pumps were placed in the tank to ensure homogenous mixing of DO in the water. During hypoxia treatment, the fish's responses were closely observed. When a fish experienced loss of equilibrium (LOE) and was unable to regain its normal posture within 10 s, the corresponding time and DO concentration were recorded. At the same time, the fish were immediately anesthetized using tricaine methanesulfonate (MS‐222), and the dorsal fins of the fish were collected and stored in anhydrous ethanol. Hypoxia tolerance phenotype was defined as the LOE time. The water temperature (22°C–23°C), salinity (26–28), and pH (7.8–8.0) of all groups were consistent during the experiment in different years.

### 2.3 DNA Extraction and Genotyping

Each group of experimental fish was sorted according to the order of LOE time, and a total of 898 samples from the top and bottom 10% of samples were subjected to DNA extraction. DNA extraction was performed using a commercial kit (Omega Bio‐Tek, USA) according to the corresponding experimental steps, and the DNA integrity and concentration were examined using 1% agarose gel electrophoresis and Nanodrop‐2000 spectrophotometer (Thermo Scientific, USA). The DNA samples passing the quality control were sent to Shijiazhuang MolBreeding Biotechnology Co., Ltd for genotyping using a 55K SNP panel of *L. crocea* [15]. This 55K panel was generated from a single marker panel using the in‐solution probe capture method by controlling sequencing depth, allowing multiple SNPs (mSNPs) surrounding each target SNP to be captured. Samples with gene deletion rates >10%, as well as SNP with genotype deletion rates >10%, minimum allele frequencies (MAF) <5%, and SNPs that did not conform to the Hardy–Weinberg equilibrium, were filtered out using the software PLINK (v. 1.90) [16]. resulting in 52 001 SNPs and 71 907 mSNPs from 884 samples (Table S2).

### 2.4 Population Structure Analysis and Selective Signal Analysis

Based on these clean SNPs, phylogenetic trees were constructed using the neighbor‐joining method via PHYLIP (v. 3.698) and visualized using Interactive Tree of Life [17]. Principal Component Analysis (PCA) was performed using GCTA (v. 1.26.0), and PCA distribution maps were drawn using the R software (v. 4.0.4) [18]. Population structure analysis was performed using Admixture (v. 1.3.0) and visualized using TBtools‐II [19]. Individual kinship matrices were plotted using the R package GAPIT (v. 3.0) [20]. Linkage disequilibrium (LD) was performed using PopLDdecay (v. 3.43) [21]. Fixation index (*F*st) and nucleotide diversity (π) were calculated using VCFtools (v. 0.1.17) in a sliding window method and selective signal analysis based on *F*st and π was performed [22]. Specifically, *F*st and π values were calculated using a 100 kb window sliding in 10 kb steps. Regions with *F*st  >  0.197 and log10 (θπ ratio) > 1.06 were considered strong signals for selective scanning.

### 2.5 WGS and SNP Calling

In the present study, an additional 600 *L. crocea* (300 DQ_1 and 300 MD_1) were subjected to hypoxia stress experiments, and their hypoxia phenotypic data were collected. DNA was extracted from 120 of these samples, and a resequencing library with a 350‐bp insert fragment size was constructed on the quality‐inspected DNA samples using the GenoBaits DNA‐seq Library Prep kit (MolBreeding Biotechnology, China). Finally, sequencing was performed using the MGI‐2000 sequencing platform of BGI with a read length of 2 × 150 bp. After sequencing, the raw reads were filtered by Fastp (v. 0.23.2) to obtain clean reads [23], and the clean reads were aligned with the reference genome of *L. crocea* (accession No. GCA_003845795.1) using BWA (v. 0.7.17) [24]. The module HaplotypeCaller of GATK (v. 4.4.0.0) was used for variation calling and counting the number of SNPs for each individual. In total, sequence data with an average of 10.55× depth, 96.75% coverage, and 15,999,230 SNPs were obtained (Table S3) [25]. The raw SNPs obtained were filtered using PLINK according to the above criteria, and finally, a total of 8 664 269 high‐quality SNPs from 120 *L. crocea* samples were obtained for further analysis.

### 2.6 GWAS

GWAS was performed on the obtained high‐quality SNP data (including SNP_chips and WGS data) and phenotypic data using mixed linear modeling with the software GEMMA (v. 0.98.1) [26], followed by Manhattan and quantile‐quantile (Q‐Q) plots using the R package CMplot (v. 3.6.0) [27]. In the GWAS, to account for potential confounding factors (e.g., body weight, body length, and sex), we included these variables as covariates in a mixed linear model to adjust for non‐genetic influences on the phenotype. To avoid false‐positive results, a modified Bonferroni multiple hypothesis test was used to define the significance thresholds; the significant association threshold and suggested association threshold at the genome‐wide level were 0.05/N and 1/N, respectively, where N is the number of SNPs used for the association analysis. Genes upstream and downstream of 100 kb of the obtained significant SNPs were scanned, and the coding sequences closest to each SNP were extracted and annotated in the NCBI database using the BLAST tool to identify potential candidate genes. The functions of these genes were further determined based on existing literature and the genomic location of the candidate genes. Haploview (v. 4.2.0) was also used to generate LD maps of SNPs flanked by significantly related SNPs as well as for haplotype analysis [28].

### 2.7 RNA Sequencing Analysis

Two hundred *L. crocea* were randomly selected from each of the DQ_1 and DQ_2 groups and transferred to indoor culture tanks for 14 d of temporary rearing. After the hypoxia tolerance traits were evaluated to confirm a significant difference (Figure S3), the two groups of fish were subjected to an acute hypoxia stress experiment. From each group, 150 selected *L. crocea* were placed in three round culture tanks (50 fish per tank), which were normally aerated for 12 h, to acclimatize the fish to the environment. Subsequently, the aeration was stopped, and nitrogen was rapidly injected into the water of the tanks, such that the DO content rapidly decreased from the normoxia state (7.0–8.0 mg/L) to 2.0 mg/L within 20 min, following which the tanks were covered with a transparent plastic film to isolate the influence of oxygen in the air [29]. The hypoxia experiment was also conducted using the aforementioned automated DO controller. Starting from the time when the DO decreased to 2.0 mg/L, three fish from each of the three parallel experimental tanks of the hypoxia‐tolerant group (T) and control group (N) were taken at 0, 6, and 48 h, immediately anesthetized using MS‐222, and then placed on ice trays to respect the livers, which were snap‐frozen with liquid nitrogen and stored at −80°C. Total RNA from the liver tissues was extracted using an RNA extraction kit (Omega Bio‐Tek, USA) and recorded as T_0 h, T_6 h, T_48 h, and N_0 h, N_6 h, N_48 h, respectively. Library construction, sequencing, and differential gene expression analysis were performed as described previously [30].

### 2.8 DNA Methylation Analysis

The samples were genotyped based on PCR (see Table S4 for primers) and Sanger sequencing, and classified into Hap1 and Hap2 based on the results of haplotype analysis. The Hap1 and Hap2 fish were subjected to hypoxia stress for 48 h according to the above method, and their genomic DNA was extracted. The qualified DNA was treated with bisulfite, followed by library construction using the DNA methylation library construction kit (Illumina, USA). The constructed libraries were constructed using the Illumina Novaseq platform for whole‐genome bisulfite sequencing (WGBS), with three biological replicates per haplotype. After the clean data were subjected to genomic comparison, MethylKit (v. 1.0.2) was used for methylation site detection and differential methylation region (DMR) analysis [31]. Subsequently, six *L. crocea* each of Hap1 and Hap2 were analyzed to detect the level of DNA methylation modification in the intron 2 region of the gene encoding lipoic acid synthase (*lias*) using BSP. Qualified genomic DNA was then subjected to bisulfite treatment and PCR amplification (Table S4) using the EZ DNA Methylation‐Gold kit (Zymo Research Corp, USA), and ten positive clones were selected for sequencing after ligation transformation of the PCR products. Sequencing results were analyzed using the BiQ Analyzer to determine DNA methylation of the intron 2 region of *lias*.

### 2.9 ATAC and CUT&Tag Sequencing

ATAC‐seq libraries amplification and purification were performed according to standard ATAC‐seq protocols [32]. In brief, liver tissues from Hap1 and Hap2 *L. crocea* treated with hypoxia were lysed on ice to isolate cell nuclei, followed by digestion of the open‐access DNA with Tn5 transposase. The digested DNA fragments were extracted and purified using a PCR purification kit. Following quality control, the DNA library was sequenced on the Illumina PE150 platform at a coverage depth of 20G per sample.

CUT&Tag libraries amplification and purification were performed according to the standard CUT&Tag protocol [33]. In brief, a cell suspension was prepared from Hap1 and Hap2 *L. crocea* liver tissue and bound to concanavalin A‐coated magnetic beads. Cell membranes were permeabilized using digitonin, followed by incubation with primary antibodies and secondary antibodies and protein A‐Tn5, and fragmentation. DNA fragments were then extracted for library construction and sequencing. The DNA library was sequenced on the Illumina PE150 platform at a coverage depth of 6G per sample. The antibodies used in the CUT&Tag protocol included H3K4me1, H3K4me3, and H3K27ac. Library preparation and sequencing for the ATAC and CUT&Tag were both performed by Frasergen Bioinformatics Co., Ltd. (Frasergen), Wuhan, China.

### 2.10 Dual‐Luciferase Reporter Assay

Based on the predicted CpG islands in the genome sequence, we divided intron 2 of *lias* into three fragments, Region1, Region2, and CGI2 (Figure S4). The sequences of the three fragments were PCR‐amplified (Table S4) with DNA from two *L. crocea* (CpG‐SNP LG1_8315503 for the two fish were G/G and A/A, respectively), inserted into the pGL3‐promoter vector, and verified using automated sequencing. The sequencing‐validated CGI2 fragments were methylated using the CpG methyltransferase M.SssI (#EM0821, Thermo Scientific, USA) and designated as mCGI2. The PGL3 plasmids and pRL‐TK control vector were co‐transfected into HEK‐293 cells using Lipofectamine 3000 (Invitrogen, USA). Luminescence was measured 24 h after transfection using the Double‐Luciferase Reporter Assay Kit (Genecreate, China). Additionally, to evaluate the prolyl hydroxylase activity of PHD, HEK293T cells were co‐transfected with ODD‐Luc‐pcDNA3 (18965, Addgene, USA) [34]. Luminescence measurements were performed in triplicate using a SpectraMax M5 (Molecular Devices, USA), and at least six biological replicates were performed.

### 2.11 Cell Culture and Hypoxia Challenge

The cells used in this experiment were the large yellow croaker fry (LYCF) cell line. The authenticity of the LYCF cell line was verified via chromosome typing, with a chromosome number of 2*n* = 48, consistent with that of *L. crocea*. The LYCF cells were cultured in L‐15 medium (Solarbio, China) supplemented with 10% fetal bovine serum (Gibco, USA) and 1% penicillin and streptomycin (Gibco, USA) at 27°C [35]. After the LYCF cells were cultured in six‐well plates, they were placed in a cellular hypoxia chamber (MIC‐101, Genodia, China) with 1% O_2_ and 99% N_2_ for 24 h.

### 2.12 Quantitative Reverse Transcription‐PCR and Chromatin Immunoprecipitation Quantitative Real‐Time PCR

Total RNA from the tissues and cells was extracted using an RNA extraction kit according to the manufacturer's protocol. Reverse transcription was then performed using the HiScript III RT SuperMix for qPCR Kit (Vazyme, China). Quantitative PCR (qPCR) was performed using the LightCycler 480 instrument (Roche, Switzerland) with SYBR Green (Vazyme, China). Three biological replicates and three technical replicates were determined for each group during the experiment, and the results were analyzed using the 2*^−ΔΔct^* method. *β‐actin* was used as the reference gene for normalization (see primers in Table S4).

ChIP‐qPCR was used to validate the immunoprecipitated DNA to assess the enrichment of the CGI2 site in the *lias* gene within MAFK. Chromatin was immunoprecipitated using anti‐MAFK (ab229766, Abcam, England), with IgG (A7016, Beyotime, China) serving as a negative control. qPCR was performed using SYBR Green Mix (Vazyme, China). The signals obtained from each immunoprecipitation were expressed as a percentage of the total input chromatin.

### 2.13 Western Blot Analysis

Total protein was extracted from the tissues and cells using radioimmunoprecipitation assay (RIPA) buffer with phenylmethanesulfonyl fluoride (PMSF; Beyotime, China). Proteins were separated using sodium dodecyl sulfate polyacrylamide gel electrophoresis and transferred to polyvinylidene fluoride membranes (Millipore, USA) using the wet‐transfer method. After blocking with 5% nonfat milk, the washed membrane was incubated with the primary antibody solution for 12 h at 4°C. After washing the membrane, it was then incubated with the secondary antibody solution for 2 h at 37°C. Immunoreactive bands were visualized using the imaging analysis system (Tanon, China). The primary antibodies included mouse monoclonal antibody against Actin (AA128, Beyotime, China), mouse monoclonal antibody against LIAS (67298‐1, ProteinTech, China), mouse monoclonal antibody against hydroxy‐HIF1α (3434, Cell Signaling Technology, USA), rabbit monoclonal antibody against PHD2 (ab226890, Abcam, England), and the HIF‐1α antibody, which was self‐manufactured by our group; the secondary antibody was the goat anti‐mouse antibody (A0192, Beyotime, China) and goat anti‐rabbit antibody (A0208, Beyotime, China). The specificity of the self‐produced antibody and commercial antibody has been validated (Figure S5). Cells were treated with cyclohexylimide (CHX; MedChemExpress, USA), and the cell protein samples treated with CHX for 0, 0.5, 1, and 2 h were collected. Protein expression levels were detected via Western blot analysis, and protein stability was assessed.

### 2.14 RNA Interference and Overexpression

The small interfering RNA (siRNA) targeting *lias* were synthesized by Genepharma Technology Co, Ltd. The sequences of gene‐specific siRNA and negative control are shown in Table S4. siRNAs were transfected into LYCF cells using Lipofectamine 6000 reagent (Beyotime, China) and cultured for 24 h followed by hypoxia challenge or normoxia culture (21% O_2_). For the overexpression experiments, *lias* and DNA methyltransferases 1 (*dnmt1*) was PCR‐amplified using specific primers (Table S4) and ligated into pcDNA3.1 to construct oe‐*lias* overexpression vectors. The plasmid was then transfected into LYCF cells using Lipofectamine 6000 reagent and cultured for 24 h followed by hypoxia or normoxia treatment.

### 2.15 Quantitative Proteomic Analysis

After collecting the LYCF cells, they were lysed using a PMSF‐containing lysis buffer, followed by sonication, washing, and quantification via the BCA assay. Subsequently, proteolytic digestion and tandem mass tag (TMT) labeling were performed, followed by liquid chromatography‐tandem mass spectrometry (LC‐MS/MS) analysis using the Vanquish Neo UHPLC system (Thermo Scientific, USA). TMT quantification and identification were performed using Proteome Discoverer software (v. 2.5) [36]. Proteins with *p* < 0.05 and |fold change (FC)| ≥ 1.5 were defined as differentially abundant proteins (DAPs) and further analyzed via GSEA enrichment analysis. The steps for transcriptomic analysis of cells are the same as in Section 2.7.

### 2.16 Metabolomic Analyses

The cells were washed with ice‐cold PBS, and a methanol‐water mixture (4:1) was added to extract the metabolites. Chloroform was then added to the samples and mixed thoroughly, followed by sonication in an ice‐water bath, and centrifugation at 4°C. Finally, the supernatant was transferred to an Agilent 7890B gas chromatography system (Agilent Technologies, USA) and a Waters ACQUITY UPLC I‐Class plus (Waters, USA) for gas chromatography‐mass spectrometry (GC‐MS) and LC‐MS/MS analysis, respectively. Raw data were imported and processed for peak detection, peak identification, and characterization using the MS‐DIAL software (v. 4.24) [37], followed by similarity matching to public databases based on retention time, mass‐to‐charge ratio, and peak area for metabolite characterization and quantification. Differential metabolites were selected with variable importance of projection values > 1.0 and *p*‐values < 0.05.

### 2.17 Quantification of Metabolites and Enzyme Activities

A mixture of methanol and 0.1% phosphoric acid (3:2) was added to the cells; the cells were then homogenized in an ice bath, sonicated at room temperature for 30 min, and then centrifuged to obtain the supernatant. The supernatant was transferred to a high‐performance liquid chromatographic system (LC‐20A, Shimadzu, Japan) to determine the contents of lipoic acid (LA) and α‐ketoglutaric acid (α‐KG). For the enzyme activity assay, the cells were washed twice with an appropriate amount of pre‐cooled PBS, and then an appropriate amount of the corresponding enzyme activity lysate was added, followed by the determination of pyruvate dehydrogenase (PDH; BC0385, Solarbio, China), α‐ketoglutarate dehydrogenase (α‐KGDH; BC0715, Solarbio, China), dihydrolipoyl transacetylase (DLAT; KT69823, DuQiao, China), and dihydrolipoamide succinyltransferase (DLST; KT69803, DuQiao, China) activities, according to the respective kit instructions.

### 2.18 RNA Interference In Vivo

The *L. crocea* (body weight 85 ± 1.85 g) used for siRNA interference experiments were obtained from the Ningbo Academy of Oceanology and Fishery, all originating from the same parental stock and belonging to the same breeding area. The *L. crocea* were maintained in a recirculating aquaculture system for scientific collective rearing (Temperature 22±1°C) for 14 d. At the start of the experiment, each fish received an intraperitoneal injection of si_*lias* (2'‐O‐methyl (OMe)+5'‐cholesterol (chol)‐modified) or si_NC (2'‐OMe+5'‐Chol‐modified) at a dose of 2 mg/kg (Table S4). After 72 h of injection, a portion of the fish were collected for siRNA silencing effect detection, while the remaining fish underwent hypoxic stress according to the experimental protocol described in Sections 2.2 and 2.7, with survival rates recorded for each individual.

## 3 Results

### 3.1 Genomic Variation and Population Structure Analysis

**FIGURE 1:**
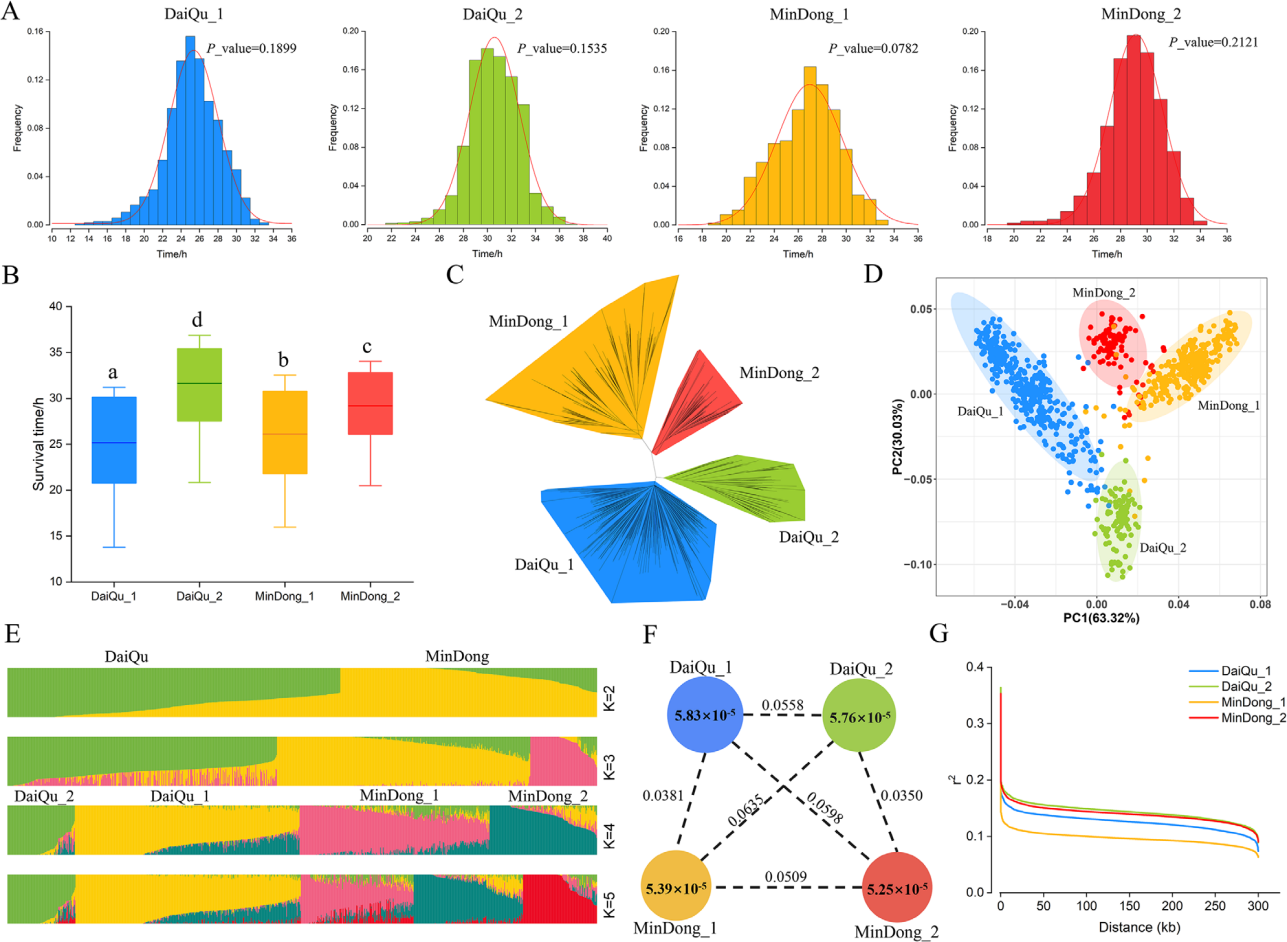
Hypoxia tolerance phenotypic data statistics and population structure analysis of *Larimichthys crocea* used in this study. (A) Histogram for mortality frequency under hypoxia stress for DaiQu_1 (blue), DaiQu_2 (green), MinDong_1 (yellow), and MinDong_2 (red) *L. crocea*. The *p*‐value for the normality test (Kolmogorov–Smirnov test) is specified in the upper right corner of the graph. (B) Mean survival time under hypoxia stress for the four populations of *L. crocea*. (C) Phylogenetic tree for all the *L. crocea* samples. (D) Principal component analysis (PCA) plot of the first two components of all *L. crocea* samples. (E) Admixture plots at ancestry numbers K = 2, 3, 4, and 5 for *L. crocea* samples. (F) Population differentiation (*F*_ST_) and nucleotide diversity (π) among the four *L. crocea* populations. The values in the circle and dotted line represent the π and *F*_ST_ values, respectively, between the two populations. (G) Linkage disequilibrium (LD) decay plot for the four populations of *L. crocea*. The X‐axis presents the physical location and Y‐axis presents the LD value (r^2^). Different letters indicate statistically significant differences at *p*  <  0.05.

This study included the phenotypic data of hypoxia tolerance from 4347 *L. crocea* belonging to four populations (DQ_1, DQ_2, MD_1, and MD_2), and genotyped 898 of them using a 55K SNP panel. A total of 123 908 SNPs from 884 fish were obtained after filtering (Figure S6). Statistical analysis of the hypoxia‐tolerant phenotyping data revealed that the hypoxia survival time of different *L. crocea* varied remarkably between 13.78 h and 36.87 h, and the mortality density curves of different populations were close to normal distribution, reflecting potential genetic diversity (Figure 1A and Table S5). In addition, the mean hypoxia survival time of the hypoxia‐selected populations (DQ_2 and MD_2) was significantly higher than that of the unselected populations (DQ_1 and MD_1), suggesting the feasibility of artificial selection for hypoxia‐tolerant traits in *L. crocea* (Figure 1B). The results of phylogenetic analysis, PCA, and kinship heat map revealed that *L. crocea* involved in genotyping were divided into four large clusters, which was basically consistent with the classification of the four populations (Figures 1C,D and Figure S7). The results of structural analysis revealed that CV error was the smallest when K = 4, indicating reasonable structural classification of the four populations (Figure 1E and Figure S8). At K = 2, a clear separation was observed between DQ and MD, indicating geographical separation of the DaiQu and MinDong groups. Notably, π value of the hypoxia‐selected populations was slightly lower than that of the unselected populations, with both populations exhibiting slight genetic differentiation (Figure 1F). *F*st and π analysis demonstrated that after hypoxia selection, the allele frequencies of these SNPs were significantly altered, with multiple regions undergoing strong selection, predominantly positive selection (Figure S9). In addition, LD analysis revealed that LD decayed faster in the selected populations compared to those in the unselected populations, reflecting the high‐intensity selection of hypoxia‐tolerant phenotypes during the selection process (Figure 1G). These results reflect the vital role of artificial hypoxia selection in the breeding process of *L. crocea*.

### 3.2 Integrated Analyses Reveal the Candidate Genes for Hypoxia Tolerance in *L. crocea*

**FIGURE 2:**
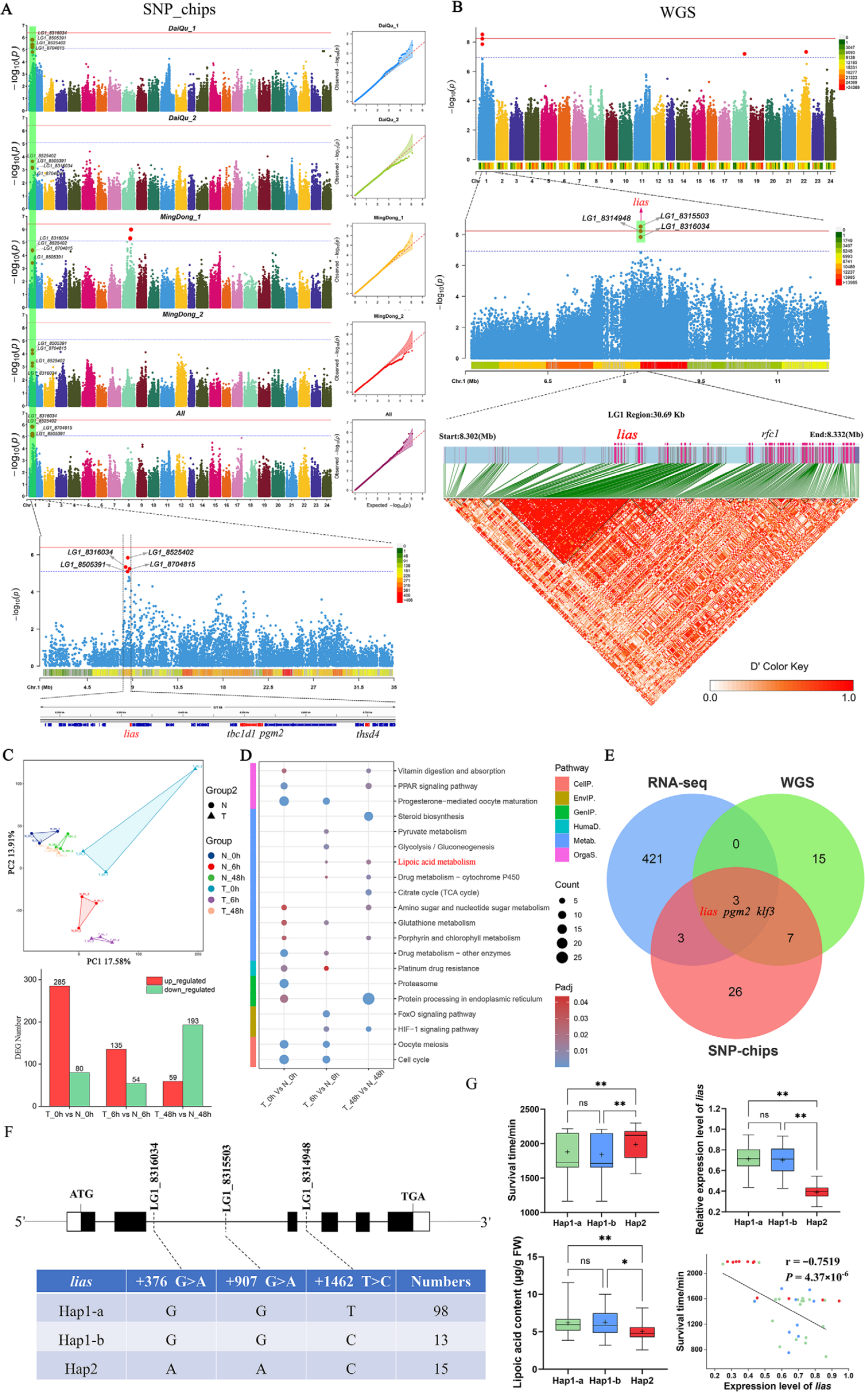
Genome‐wide association analysis (GWAS) of hypoxia tolerance, transcriptome analysis, and haplotype analysis of lipoic acid synthetase (*lias*) in *Larimichthys crocea*. (A) Manhattan plot and quantile‐quantile plot of GWAS for hypoxia tolerance from four *L. crocea* populations with SNP‐chip data. Red and blue lines correspond to genome‐wide significance (−log_10_(*P*) = 6.39) and significant suggestive association level (−log_10_(*P*) = 5.09) thresholds, respectively. Red and blue boxes, genes. (B) Manhattan plot of GWAS for hypoxia tolerance and linkage disequilibrium (LD) plot for a significant SNP in the 8.302–8.322 Mb region of chr 1 with whole‐genome resequencing data. Red and blue lines correspond to genome‐wide significance (−log_10_(*P*) = 8.23) and significant suggestive association level (−log_10_(*P*) = 6.93) thresholds, respectively. The depth of red color in the square increases with the degree of linkage and D^’^ value. (C) PCA plot of RNA‐sequencing samples and histograms of differentially expressed genes (DEGs) between hypoxia‐tolerant (T) and control (N) *L. crocea* under hypoxia stress. Red and green dots indicate significantly upregulated and downregulated DEGs, respectively. (D) Top 20 significantly enriched Kyoto Encyclopedia of Genes and Genomes (KEGG) pathways for DEGs. (E) Venn diagrams of candidate genes based on SNP_chips, WGS, and RNA‐seq. (F) Gene structure and haplotype analysis of *lias*. Black boxes represent exonic regions and solid black lines represent intronic regions. (G) Box plots of survival time, *lias* expression and lipoic acid content for different haplotypes under hypoxia stress, and correlation analysis between lipoic acid expression and survival time. Data are presented as the mean ± standard deviation (SD) of three independent experiments. ∗, *p* < 0.05; ∗∗, *p* < 0.01.

To identify the key genes regulating hypoxia tolerance in *L. crocea*, we performed GWAS analysis on the 884 genotyped samples, which resulted in the identification of four significant SNPs on chromosome 1 (Figure 2A and Table S6). Moreover, GWAS findings for each of the four populations demonstrated the stability of these SNPs at or near significant levels. According to the annotation of the reference genome, these four SNPs were distributed over a 400 kb genomic region and included 12 protein‐coding genes. To exclude the presence of other potential critical SNPs not included in the SNP_chips, we repeated the hypoxia stress experiment and WGS on an additional 120 *L. crocea*. After filtering, a total of 8,664,269 high‐quality SNPs from 120 *L. crocea* were obtained for GWAS (Figure S10A–C). Subsequently, GWAS analysis revealed a total of five significant SNPs and a peak on chromosome 1, where three SNPs were located in high LD (r^2^ > 0.9) (Figure 2B, Figure S10D, and Table S7). Annotation of the genes in the vicinity of these three SNPs revealed that all of them were located on the gene encoding lipoic acid synthase (*lias*). In addition, transcriptomic analysis of hypoxia‐tolerant and hypoxia‐sensitive *L. crocea* under hypoxia stress revealed a total of 428 differentially expressed genes (DEGs) (Figure 2C, Table S8, and Figure S11). Kyoto Encyclopedia of Genes and Genomes (KEGG) analysis of the DEGs resulted in the identification of multiple hypoxia tolerance‐related pathways, including the HIF‐1 signaling pathway and the lipoic acid metabolic pathway (Figure 2D). Overlapping analysis of these DEGs with candidate genes detected using SNP_chips and WGS (Tables S9–S11) identified a total of three candidate genes, including *lias* (Figure 2E). Therefore, *lias* was considered as a candidate gene to regulate hypoxia tolerance in *L. crocea*.

Lias is an important metabolic enzyme in living organisms, and in mammals, its primarily plays a role in LA synthesis. Multiple comparisons of the amino acid sequences and phylogenetic tree analysis (Figure S12 and Table S12) revealed that Lias is relatively more conserved during species evolution, and the similarity of proteins homologous to Lias was more than 70% from invertebrates to humans, which predicts that its function may be similar to that in mammals, which are both involved in alpha‐lipoic acid synthesis (Figure S13). *lias* is expressed in various tissues of *L. crocea*, with relatively high expression levels in the gonads, liver, and brain (Figure S14). However, to our knowledge, its involvement in regulating hypoxia tolerance in *L. crocea* has not been investigated. Based on the three significant SNPs located in the second and third intron regions of *lias* identified by GWAS analysis, the following three main haplotypes existed in the *L. crocea* population: Hap1‐a, Hap1‐b, and Hap2 (Figure 2F). Further analysis revealed no significant difference in hypoxia survival time between Hap1‐a and Hap1‐b, whereas the hypoxia survival time of Hap2 was significantly higher than that of Hap1‐a and Hap1‐b (Figure 2G). Hap2 *L. crocea*, however, exhibited significantly lower *lias* expression and LA content than those in Hap1‐a and Hap1‐b. We randomly selected three haplotypes from another *L. crocea* population for hypoxia stress and observed a negative correlation between the *lias* expression level and hypoxia survival time. The above results suggest that variation in the intronic region of *lias* may play an important role in the negative regulation of hypoxia tolerance in *L. crocea*.

### 3.3 CpG‐SNPs in Intron 2 Affect the Transcriptional Activity of *lias* via Methylation

**FIGURE 3:**
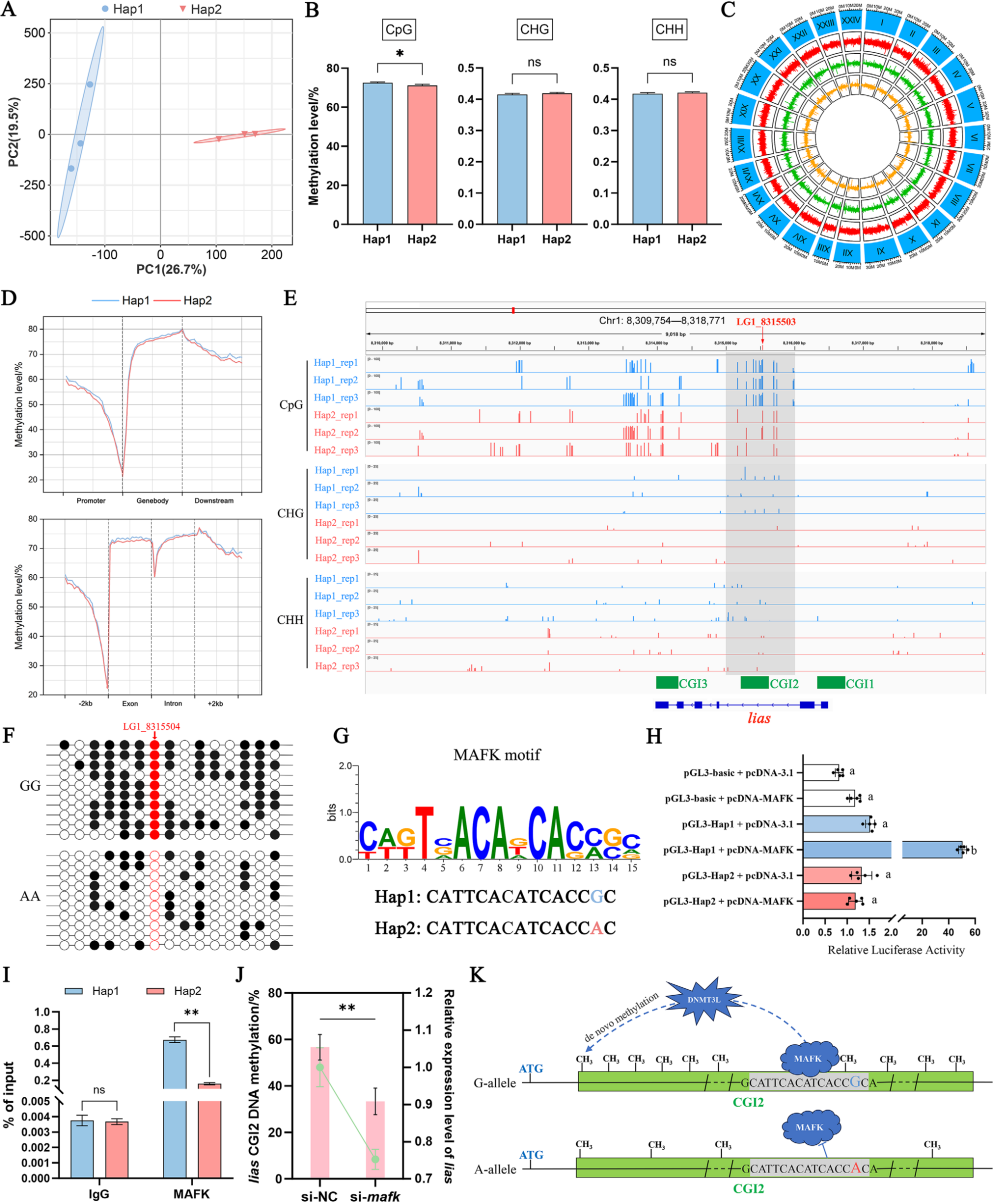
CpG‐SNP leads to methylation differences in CGI2 region of *lias* gene in *Larimichthys crocea*. (A) PCA of Hap1 and Hap2 whole‐genome bisulfite‐sequencing (WGBS) samples. (B) Methylation levels of CpG, CHG, and CHH in the Hap1 and Hap2 groups. (C) Circle plot for the genome‐wide distribution of different types of differentially methylated regions (DMRs) between Hap1 and Hap2. Distribution of CpG‐type DMRs, CHG‐type DMRs, and CHH‐type DMRs on individual chromosomes, in order from the inside out. (D) Methylation levels of CpG, CHG, and CHH in different gene structural regions of the Hap1 and Hap2 groups. (E) Distribution of different types of methylation sites on *lias* determined via Integrated Genome Browser view. The gray area represents a DMR; the green boxes represent CpG islands (Location of CGI1, CGI2, and CGI3 in chromosome 1: 8216649–8316299, 8315640‐8315434, and 8314475‐8314330). (F) Schematic diagram of different CpG methylation in the intron 2 of *lias*. Open circles and solid circles represent unmethylated and methylated CpG dinucleotides, respectively. Schematic diagram of different CpG methylation in the second intron of *lias*. Open circles and solid circles represent unmethylated and methylated CpG dinucleotides, respectively. Each row of circles represents a sequencing clone, and each column represents a CpG site. (G) Motif analysis of LG1_8315503. (H) Detection of binding between LG1_8315503 and MAFK based on dual luciferase reporter assay. Data were shown as the mean ± SD, *n*  =  6 biologically independent samples. (I) The ChIP‐qPCR experiment demonstrated the quantification of the *lias* intron 2 amplicon, which was immunoprecipitated with the MAFK antibody and then subjected to PCR amplification of the DNA fragment. ChIP‐qPCR with each of the antibodies and normal rabbit IgG was performed using four biological replicates for each genotype. (J) Effects of *mafk* interference on the methylation levels of the *lias* CGI2 region and on mRNA expression in Hap1 genotyped cells. The bar chart represents methylation levels, and the scatter plot represents mRNA levels. (K) Epigenetic‐dependent regulatory mechanism of LG1_8315503 in *lias* expression. ∗, *p* < 0.05; ∗∗, *p* < 0.01. Different letters indicate statistically significant differences at *p* < 0.05.

To further investigate how the above significant SNPs affect *lias* expression, we subjected both Hap1 and Hap2 *L. crocea* to hypoxia stress and WGBS of their DNA. Each haplotype was sequenced using three biological replicates, and approximately 80% of the reads in each sample were successfully aligned to the reference genome, with an average bisulfite conversion rate of 99.7%; PCA revealed that Hap1 and Hap2 each formed a separate cluster (Figure 3A and Table S13). Sequencing results revealed that the average CpG level of Hap1 *L. crocea* was significantly higher than that of Hap2, while there was no significant difference in CHG and CHH methylation levels (Figure 3B). In addition, the methylation levels of CpG islands (CGI) and their flanking regions in Hap1 were also significantly higher than those in Hap2. Next, we examined the DMR of the two haplotype samples and detected a total of 93,577 DMRs (Figure 3C,D and Figure S15). Interestingly, we detected a DMR (Fold Change = −17.6) in the intron 2 region of *lias*, and the methylation levels of CpG, CHG, and CHH were higher in Hap1 than those in Hap2; the majority of this DMR was located in the CGI2 region (Figure 3E). Hence, we presumed that the methylation level of CGI2 on the intron 2 of *lias* potentially affects the transcriptional activity of *lias*, which subsequently affects gene expression.

To confirm this hypothesis, we examined the methylation level of *lias* CGI2 in six fishes each of Hap1 and Hap2 under hypoxia, and determined the average methylation level of 15 CpG sites as the final value. The results revealed that the average methylation level of Hap1 type was 55.33%, which was significantly higher than that of Hap2 type (24.66%) (Figure 3F). Moreover, the C site on the left side of LG1_8315503 of Hap1 was consistently methylated, while Hap2 was consistently unmethylated, and the variation of this site formed a CpG‐SNP (Figure S16). Next, we predicted the binding of transcription factors (TFs) at this SNP, where MAFK was identified as one of the TFs (Figure 3G and Table S14). To validate this interaction, luciferase reporter assays revealed that luciferase activity with Hap1 allele was significantly higher than that of the control group (transfected with the pcDNA‐3.1 empty vector). However, there was no significant change in luciferase activity with Hap2 allele (Figure 3H). The ChIP‐qPCR results also showed that the MAFK enrichment levels were significantly higher in Hap1 than in Hap2 (Figure 3I). Furthermore, we conducted a loss‐of‐function experiment: we knocked down *mafk* in Hap1‐genotype cells and subsequently measured methylation levels in the CGI2 region and *lias* mRNA expression levels. The results showed that both methylation levels and *lias* mRNA expression levels were significantly reduced (Figure 3J). A recent study defining the binding profile of TFs to DNMT, which are enzymes responsible for maintaining DNA methylation or *de novo* deposition, has identified the interaction of MAFK with DNMT3L, which is an enzyme involved in the *de novo* methylation of DNA through interaction with DNMT3A and DNMT3B. This interaction can both activate and inhibit the action of DNMT3L and represents a potential mechanism through which the G allele promotes CGI2 *de novo* methylation under hypoxia stress. The above results indicate that the motif sequence hosting the G allele at LG1_8315503 can bind to MAFK to promote the *de novo* and subsequent methylation of CGI2. In contrast, low CGI2 methylation is associated with the minor A allele of CpG‐SNP (Figure 3K).

**FIGURE 4:**
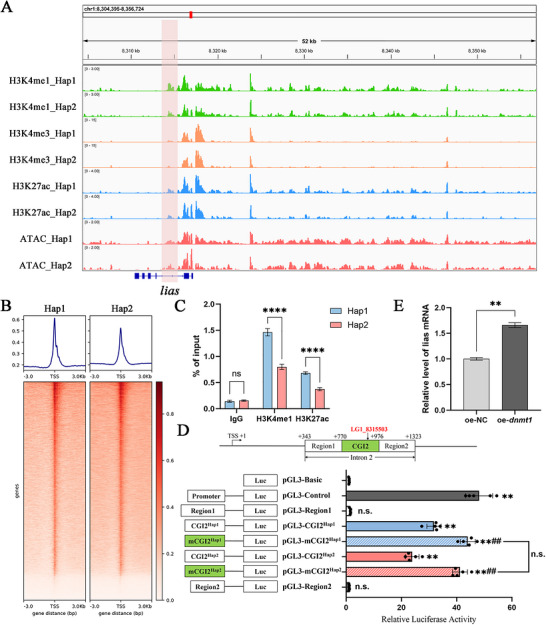
Differences in allelic methylation at LG1_8315503 result in variations in the enhancer activity of *lias* in *Larimichthys crocea*. (A) Integrative Genomics Viewer (IGV) plot showing the CUT&Tag and ATAC‐seq tracks for the *lias* gene and its upstream/downstream genes in liver tissues of *L. crocea* with Hap1 and Hap2 genotypes under hypoxia stress. (B) Profile plot and heatmap of ATAC‐seq signals around 3 Kb of the transcriptional start site (TSS) of Hap1 and Hap2 *L. crocea* under hypoxia stress. (C) Chip‐qPCR verification of Hap1 and Hap2 genotypes of *L. crocea* under hypoxia stress. (D) Effect of different regions of *lias* intron 2 region on transcriptional activity as determined using the dual luciferase reporter assay. Data are presented as the mean ± SD of six independent experiments. Significant differences from pGL3‐basic are indicated by asterisks (**, *p* < 0.01). Significant differences from the group of methylated region in the same region are indicated by pound sign (##, *p* < 0.01). (E) Effect of dnmt1 overexpression on lias mRNA expression in Hap1 genotyped cells.

To investigate how hypermethylation of CGI2 affects the gene expression of *lias*, we examined the histone modification profile of this region using CUT&Tag data from *L. crocea* liver tissue (Table S13). In general, active enhancers are marked by H3K4me1 and H3K27ac. Based on the analysis, the second intron region of the *lias* where the CpG‐SNP is located exhibits high enrichment for the histone modifications H3K4me1 and H3K27ac, suggesting the presence of a potential enhancer in this region, with enrichment being greater for Hap1 than for Hap2 (Figure 4A and Figure S17). Furthermore, based on ATAC‐seq data for Hap1 and Hap2 under hypoxic conditions, Hap1 exhibits higher chromatin accessibility, leading to its increased transcriptional activity (Figure 4B). Chip‐qPCR results also indicated that Hap1 has higher enhancer activity than Hap2 (Figure 4C). To assess the effect of methylation on enhancer activity, we inserted Region1, Region2, and different haplotypes of CGI2 into the pGL3 promoter vector, and the constructed plasmids were transfected into cells to assess the transcriptional activity. The results showed that the transcriptional activity of unmethylated CGI2^Hap1^ was significantly higher than that of CGI2^Hap2^, while the transcriptional activity of methylated Hap2 (mCGI2^Hap2^) was not significantly different from that of methylated Hap1(mCGI2^Hap1^), and was significantly higher than that of unmethylated Hap2 (CGI2^Hap2^) (Figure 4D). Additionally, we overexpressed DNA methyltransferase 1 (*dnmt1*) in genotyped cells, and the expression level of *lias* was significantly upregulated (Figure 4E). This indicates that the G allele is consistently associated with high methylation of the CGI2 region; when higher methylation is present, enhancer activity is released, leading to the activation of *lias* gene expression. Conversely, reduced methylation in the CGI2 region is associated with the minor A allele of the CpG‐SNP, which inhibits enhancer activity and gene expression.

### 3.4 *lias* Affects Hypoxia Tolerance of *L. crocea* by Regulating HIF‐1α

**FIGURE 5:**
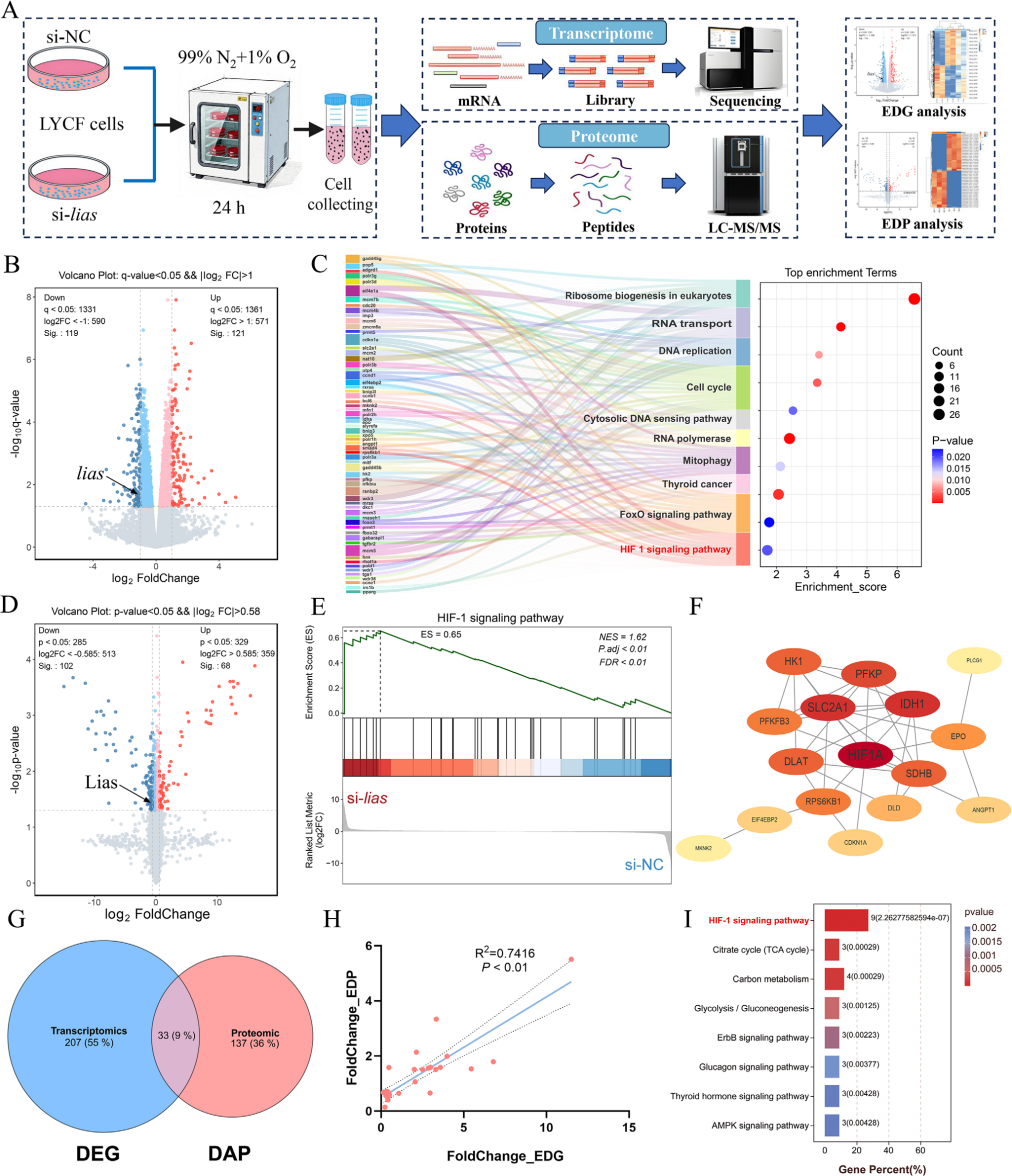
Transcriptomics and proteomics analyses reveal a close association between *lias* and HIF‐1α. (A) Schematic diagram of experimental design for sample preparation and subsequent transcriptomic and proteomic analyses. (B) Volcano diagram of differentially expressed genes (DEGs) in transcriptomic analysis. Red dots were upregulated, and blue dots were downregulated. (C) Sankey diagram summarizing the KEGG enrichment analysis results for DEGs. The left side represents the DEGs, the right side represents the significantly enriched pathways, and the branches indicate the source of DEGs for each pathway. (D) Volcano diagram of differentially abundant proteins (DAPs) in proteomics analysis. (E) GSEA analysis of DAPs indicates significant upregulation of the HIF‐1 signaling pathway. (F) Potential protein–protein interactions (PPI) among selected proteins from the GSEA analysis visualized via STRING database. (G) Venn diagram of DEGs and DAPs. (H) Spearman correlation scatter plot of 33 genes common to both DEGs and DAPs, showing moderate correlation (R^2^ = 0.7416). (I) KEGG enrichment analysis for the 33 genes common to both DEGs and DAPs.

To investigate how *lias* influences the hypoxia tolerance of *L. crocea*, we performed high‐throughput mRNA sequencing and protein sequencing in LYCF cells transfected with *lias* siRNA and subjected to hypoxic treatment (Figure 5A and Table S8). Transcriptome sequencing revealed that DEGs were significantly enriched in the HIF‐1 signaling pathway, with 17 genes showing significant differential expression identified within this pathway (Figure 5B,C and Figure S18A–C). Subsequent GSEA analysis of DAP in the proteome revealed that the HIF‐1 signaling pathway was significantly activated in the si_*lias* group compared to the si_NC group (Figure 5D,E and Figure S18D–F). Protein‐protein interaction (PPI) network analysis demonstrated that HIF‐1α exhibited a core role with high confidence in the interaction network (Figure 5F). Further integrated analysis of proteome and transcriptome data identified differentially expressed molecules with common expression trends (Table S15). KEGG enrichment analysis confirmed that the HIF‐1 signaling pathway remained the most significantly enriched pathway (Figure 5G–I). Collectively, these results suggest that *lias* may potentially influence the response process of *L. crocea* under hypoxia stress by regulating HIF‐1α.

**FIGURE 6:**
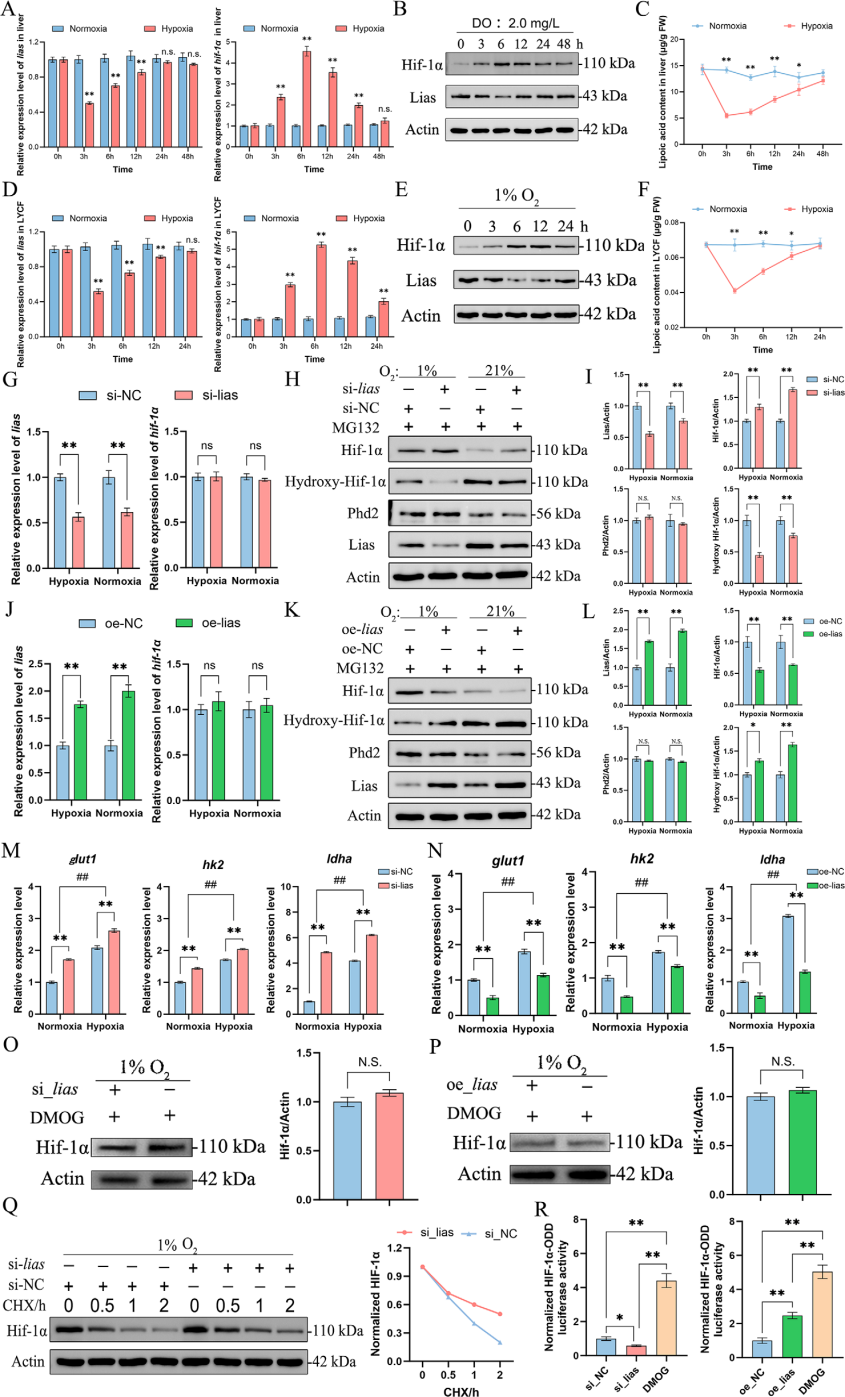
Lias regulates Hif‐1α by affecting PHD activity. (A,B) The hepatic expression of *lias* and *hif‐1α* mRNA and protein in *L. crocea* under hypoxia stress. (C) Changes in the hepatic lipoic acid content in *L. crocea* under hypoxia stress. (D) (E) The *lias* and *hif‐1α* mRNA and protein expressions in LYCF cells of *L. crocea* under hypoxia stress. (F) Changes in the lipoic acid content in LYCF cells of *L. crocea* under hypoxia stress. (G) Changes in *lias* and *hif‐1α* mRNA expression in LYCF cells in si‐NC and si‐*lias* under hypoxia and normoxia. (H) (I) Western blot analysis showing the expression of proteins of Lias, Phd2, Hif‐1α and hydroxylated Hif‐1α in si‐NC and si‐*lias* under hypoxia and normoxia. (J) Changes in *lias* and *hif‐1α* mRNA expression in LYCF cells in oe‐NC and oe‐*lias* under hypoxia and normoxia. (K) (L) Western blot analysis showing the protein expressions of Lias, Phd2, Hif‐1α and hydroxylated Hif‐1α in oe‐NC and oe‐*lias* under hypoxia and normoxia. (M) (N) The expression of Hif‐1α downstream target genes (*glut1*, *hk2*, and *ldha*) in si‐NC, si‐*lias*, oe‐NC, and oe‐*lias* under hypoxia and normoxia. *glut1*, glucose transporter type 1; *hk2*, hexokinase 2; *ldha*, lactate dehydrogenase A chain. (O) (P) Changes in Hif‐1α protein expression in LYCF cells treated with the PHD inhibitor dimethyloxalyglycine (DMOG), interference, or overexpression under hypoxic conditions. (Q) Immunoblotting analysis of LYCF cells was performed to detect the effects of interfering or overexpressing *lias* on Hif‐1α protein accumulation after cyclohexylcarboxamide (CHX) treatment (0–4 h). (R) Effects of siRNA interference or overexpression of Lias on PHD activity in 293T cells transfected with luciferase reporter. To account for transfection efficiency and values in control cells (n = 6), HIF‐1α‐ODD firefly luciferase activity was normalized to Renilla luciferase activity. DMOG‐treated cells served as positive controls. * *p* < 0.05, ** *p* < 0.01 compared to control group; # *p* < 0.05, ## *p* < 0.01 compared to normoxia group.

To verify the above conjecture, we first analyzed changes in the expressions of *lias* and *hif‐1*α under hypoxia stress. The results revealed that the hepatic mRNA and protein expression levels of *lias* in *L. crocea* decreased following hypoxia stress (Figure 6A,B and Figure S19A), accompanied by a significant decrease in the LA content (Figure 6C). Consistent with data from previous studies [38], hepatic mRNA and protein expression levels of *hif‐1*α levels increased significantly in *L. crocea*. Changes in the expression levels of *lias* and *hif‐1*α in LYCF cells were consistent with the trends observed in the liver (Figure 6D–F and Figure S19B). Next, we measured the mRNA and protein expression levels of Hif‐1α in LYCF cells subjected to interference or overexpression treatments under hypoxia and normoxia. We observed that, compared with the control group, HIF‐1α mRNA expression in the si‐*lias* group and the oe‐*lias* group showed no significant change under hypoxia stress; whereas Hif‐1α protein expression increased by 0.29‐fold and decreased by 0.45‐fold in the si‐*lias* and oe‐*lias* groups, respectively (Figure 6G–L). Similarly, under normoxia conditions, interference significantly upregulates Hif‐1α protein expression, whereas overexpression significantly downregulates Hif‐1α protein expression. Moreover, the downstream target genes of Hif‐1α, including *glut1*, *hk2* and *ldha*, were also significantly altered (Figure 6M,N). However, under conditions where *hif‐1α* was interfered, re‐evaluating the effects of *lias* overexpression or interference on downstream genes revealed that the expression changes in these genes were not significant (Figure S19C,D). Based on these observations, we propose that *lias* may regulate Hif‐1α protein by affecting its degradation rather than its transcription activity. In order to further understand how *lias* can promote the degradation of Hif‐1α, we also used a specific antibody to identify the hydroxylated HIF‐1α to determine the level of hydroxylated Hif‐1α. The results showed that interference with *lias* inhibited the hydroxylation of Hif‐1α under both hypoxic and normoxic conditions, while overexpression increased the hydroxylation (Figure 6G–L). Subsequently, we treated LYCF cells with dimethyloxalyglycine (DMOG, a known prolyl hydroxylase inhibitor) to inhibit Phd activity, followed by interference or overexpression to assess Hif‐1α changes in the presence of DMOG. We found that *lias* exerted no additional effect on Hif‐1α levels when PHD activity was inhibited, implicating that *lias* and DMOG acted on the same pathway (Figure 6O,P). Then we compared the half‐life of Hif‐1α in cells with interference or overexpression. The half‐life of Hif‐1α is between 1 and 2 h without interference, but si‐*lias* prolongs the half‐life of Hif‐1α to more than 2 h, and overexpression shortens it (Figure 6Q). In the classical model of HIF‐1α degradation, the key proline of PHDs hydroxylation is located in the ODD domain of HIF‐1α, which acts as the binding site of VHL ubiquitin ligase complex that mediates ubiquitination and proteasome degradation of HIF‐1α. Therefore, we transfected a luciferase reporter construct (ODD‐luciferase) with HIF‐1α ODD domain fused with luciferase in 293T cells, so that the stability of luciferase expression was regulated by PHD [34]. Using this reporter construct, we found that si‐*lias* stimulation increased the expression of luciferase, which further indicated that interference with *lias* directly inhibited the enzymatic activity of PHD (Figure 6R). In addition, we did not observe the change of Phd2 protein level, which indicated that *lias* could increase the protein level of Hif‐1α by inhibiting the enzyme activity of Phd2 instead of changing the cellular enzyme level. Altogether, we concluded that *lias* promotes the accumulation of Hif‐1α by inhibiting the hydroxylation degradation mediated by Phd2.

**FIGURE 7:**
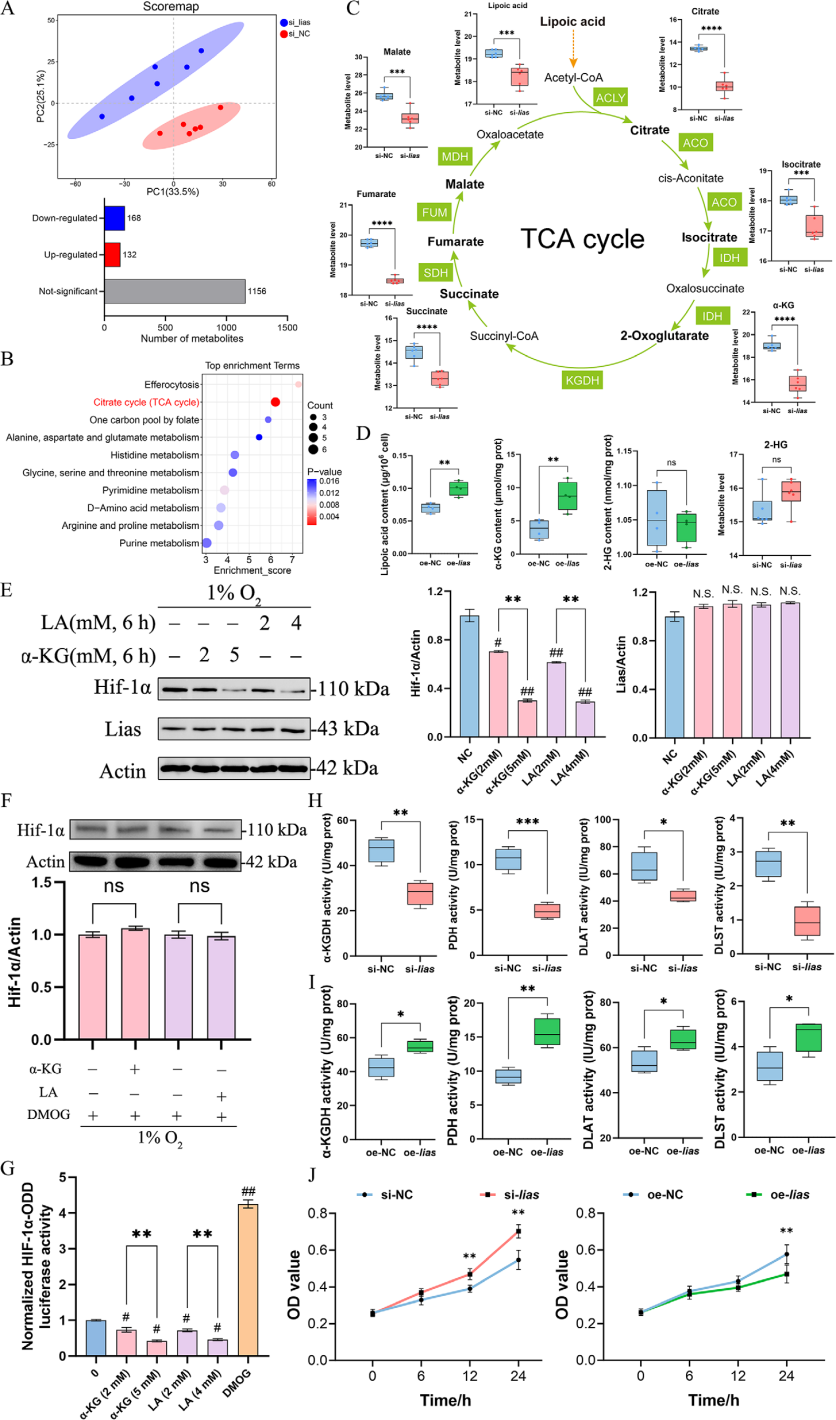
Lias regulates the α‐KG concentration by affecting the lipoylation of DLAT/DLST. (A) Scatterplot of orthogonal partial least squares‐discriminant analysis model scores and histogram of differential metabolites for the si‐NC and si‐*lias* group. (B) KEGG enrichment analysis of differential metabolites between the si‐NC and si‐*lias* groups. (C) The total metabolite levels of lipoic acid, malate, fumartae, citrate, α‐KG, succinate, and isocitrate in the si‐NC and si‐*lias* groups determined using GC‐MS and LC‐MS/MS. (D) The total metabolite levels of lipoic acid, α‐KG, and 2‐HG interfered and overexpressed cells determined using HPLC and GC‐MS. (E) Changes in the protein expression levels of Lias and Hif‐1α in LYCF cells by adding α‐KG or LA under hypoxia conditions. (F) Changes in Hif‐1α protein expression in LYCF cells treated with the DMOG, α‐KG, or LA under hypoxia conditions. (G) Effects of α‐KG or LA treatment on PHD activity in 293T cells transfected with luciferase reporter. To account for transfection efficiency and values in control cells (n = 6), HIF‐1α‐ODD firefly luciferase activity was normalized to Renilla luciferase activity. DMOG‐treated cells served as positive controls. (H) (I) Changes in PDH, α‐KGDH, DLAT, and DLST activities in si‐NC, si‐*lias*, oe‐NC, and oe‐*lias* groups under hypoxia stress. PDH, pyruvate dehydrogenase; α‐KGDH, α‐ketoglutarate dehydrogenase; DLAT, dihydrolipoyl transacetylase; DLST, dihydrolipoamide succinyltransferase. (J) Changes in proliferative activity in the si‐NC, si‐*lias*, oe‐NC, and oe‐*lias* groups under hypoxia stress. * *p* < 0.05, ** *p* < 0.01 compared to control group.

Since PHD is a member of the α‐KG‐dependent dioxygenase family and its activity is regulated by both oxygen concentration and α‐KG, HIF‐1α regulation by *lias* may be a consequence of the metabolic inhibition of PHD. To test this speculation, we performed GC‐MS and LC‐MS/MS analysis for the metabolites in LYCF cells after siRNA under hypoxia stress (Figure 7A and Figure S19E–H). The results revealed that the tricarboxylic acid (TCA) cycle‐related metabolites were greatly affected in the si‐*lias* group compared with those in the si‐NC group, with significantly lower levels of malate, fumarate, citrate, succinate, isocitrate, and α‐KG (Figure 7B,C). Moreover, the α‐KG content in the overexpression group was significantly higher than that in the control group (Figure 7D). Although previous studies have shown that α‐KG is converted into 2‐HG to influence PHD activity, neither interference nor overexpression altered 2‐HG levels in LYCF cells in this experiment. Therefore, we conclude that *lias* directly affects PHD activity by influencing α‐KG. Subsequently, we added α‐KG and lipoic acid (LA) to the culture medium after *lias* interference; consequently, both α‐KG and LA reduced the levels of HIF‐1α in a dose‐dependent manner (Figure 7E). Next, we found that the addition of α‐KG or LA had no additional effect on Hif‐1α levels in the presence of DMOG (Figure 7F). Furthermore, results from ODD‐luciferase reporter assays supported that α‐KG or LA directly promotes PHD activity on the ODD hydroxylation (Figure 7G). Collectively, we conclude that α‐KG or LA promotes HIF‐1α accumulation by inhibiting PHD‐mediated HIF‐1α degradation. Since the lipoylation proteins PDH and α‐KGDH are key enzymes in the TCA cycle, and lipoylation modifications occur in DLAT, the E2 subunit of PDH, and in DLST, the DLST of α‐KGDH, we investigated whether *lias* regulates the α‐KG concentration by modifying the activity of TCA cycle enzymes via lipoylation modifications. The results revealed that the activities of PDH, α‐KGDH, DLAT, and DLST were significantly decreased in the si‐*lias* group compared with those in the si‐NC group, and were significantly increased in the oe‐*lias* group compared with that in the oe‐NC group (Figure 7H,I). In addition, the activities of two other LA‐associated enzymes (Glycine cleavage system H protein, GCSH; Branched‐chain alpha‐ketoacid dehydrogenase kinase, BCKDH) were significantly altered upon interference or overexpression, yet the mechanism by which they affect HIF‐1α is unknown (Figure S19I). Following this, the proliferative activity of LYCF cells after interference or overexpression under hypoxia stress was examined using CCK8; the results revealed that the si‐*lias* group significantly promoted the proliferation of LYCF cells under hypoxia, while the oe‐*lias* group had a significant inhibitory effect on cell proliferation (Figure 7J). The above results indicated that *lias* downregulation could reduce DLAT and DLST lipoylation levels, which in turn decreased the α‐KG content through the TCA cycle, inhibited PHD activity and stabilized HIF‐1α protein levels, consequently inducing the activation and expression of more downstream target genes.

### 3.5 Interference With *lias* in *L. crocea* Facilitates Hypoxia Tolerance

**FIGURE 8:**
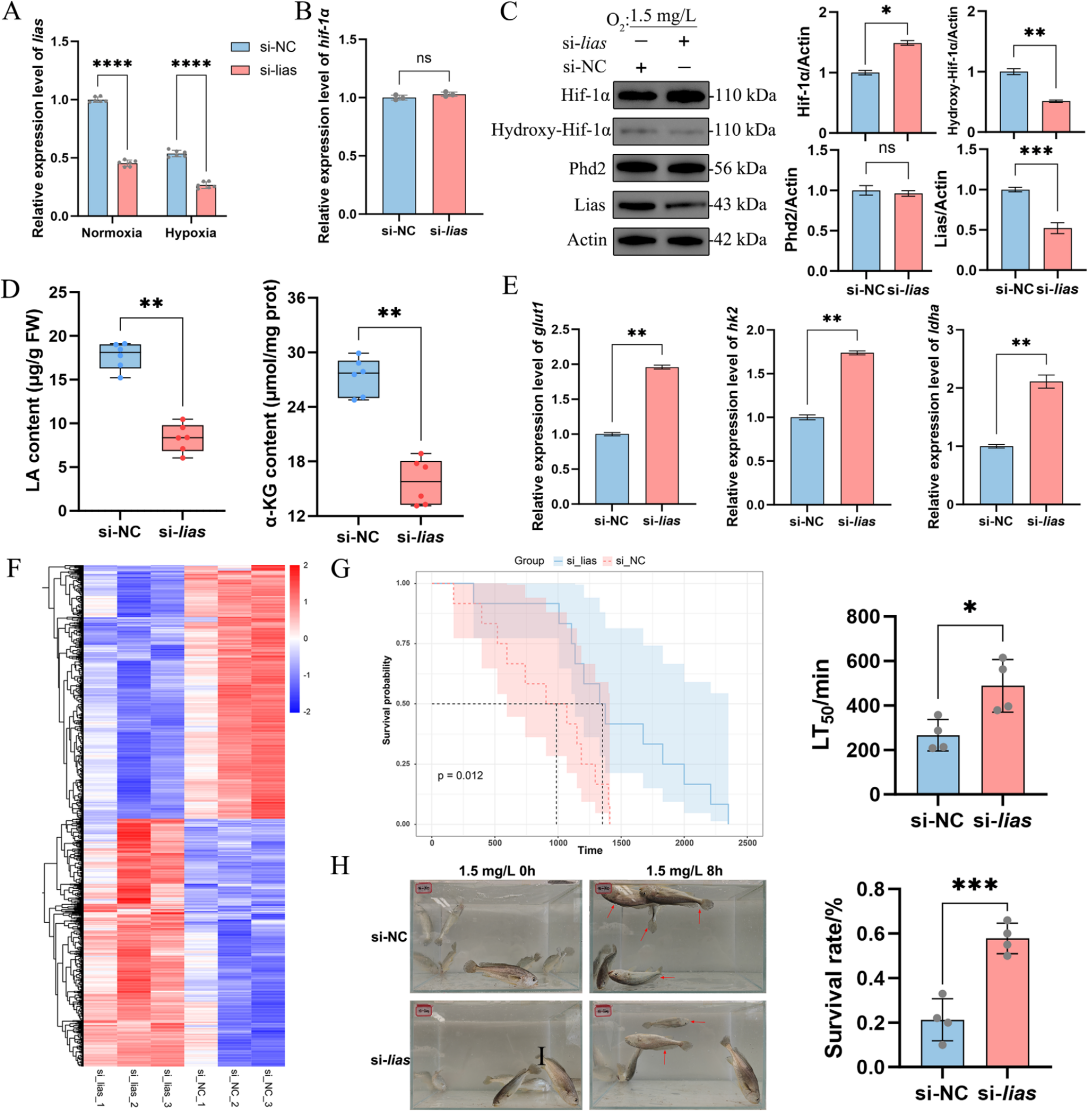
Disruption of *lias* in *L. crocea* facilitates hypoxia tolerance. (A,B) qPCR analysis showing the expression of mRNA of *lias* and *hif‐1α* in si‐NC and si‐*lias* group under hypoxia and normoxia. (C) Western blot analysis showing the expression of proteins of Lias, Phd2, Hif‐1α, and hydroxylated Hif‐1α in si‐NC and si‐*lias* under hypoxia. (D) The total metabolite levels of lipoic acid and α‐KG in si‐NC and si‐*lias* under hypoxia. (E) qPCR analysis showing the expression of mRNA of *glut1, hk2, and ldha* in si‐NC and si‐*lias* group under hypoxia. (F) Hepatic transcriptome analysis of the si_lias and si_NC groups under hypoxic stress. (G) The survival analysis and survival rate of si‐NC and si‐*lias* group under gradient oxygen reduction. (H) The field experiment diagrams and half‐lethal time statistics of the si‐NC and si‐*lias* group under acute hypoxia stress (DO = 1.5 mg/L). Red arrows, dying *L. crocea*. * *p* < 0.05, ** *p* < 0.01 compared to control group.

To determine the physiological role of *lias* in enhancing HIF‐1α activity, we conducted *lias*‐siRNA experiments in *L. crocea*. Quantitative results showed that *lias*‐siRNA significantly reduced *lias* expression under both normoxic and hypoxic conditions (Figure 8A). Consistent with observations in cell culture systems, mRNA expression of *hif‐1α* showed no significant difference under hypoxia, while protein expression was significantly upregulated, and the hydroxylated HIF‐1α was also significantly reduced after interference (Figures 8B,C). Metabolite detection also showed that *lias* affected the activity of PHD by affecting α‐KG (Figure 8D). Additionally, expression of Hif‐1α downstream target genes *glut1, hk2*, and *ldha* was significantly elevated in si_*lias* group compared to si_NC group (Figure 8E). Transcriptome sequencing also revealed significant differences in gene transcription between the two groups of fish under hypoxic conditions (Figure 8F). We subjected both si_*lias* and si_NC *L. crocea* to hypoxia stress. Results showed that si_*lias* survival rates under hypoxia ranged from 50% to 66%, significantly higher than the 10% to 33% observed in the si_NC group (Figure 8G). The half‐lethal time further indicated that si_lias *L. crocea* exhibited greater tolerance to hypoxia (Figure 8H).

### 3.6 Lias Potentially Improves Hypoxia Tolerance in *L. crocea*

**FIGURE 9:**
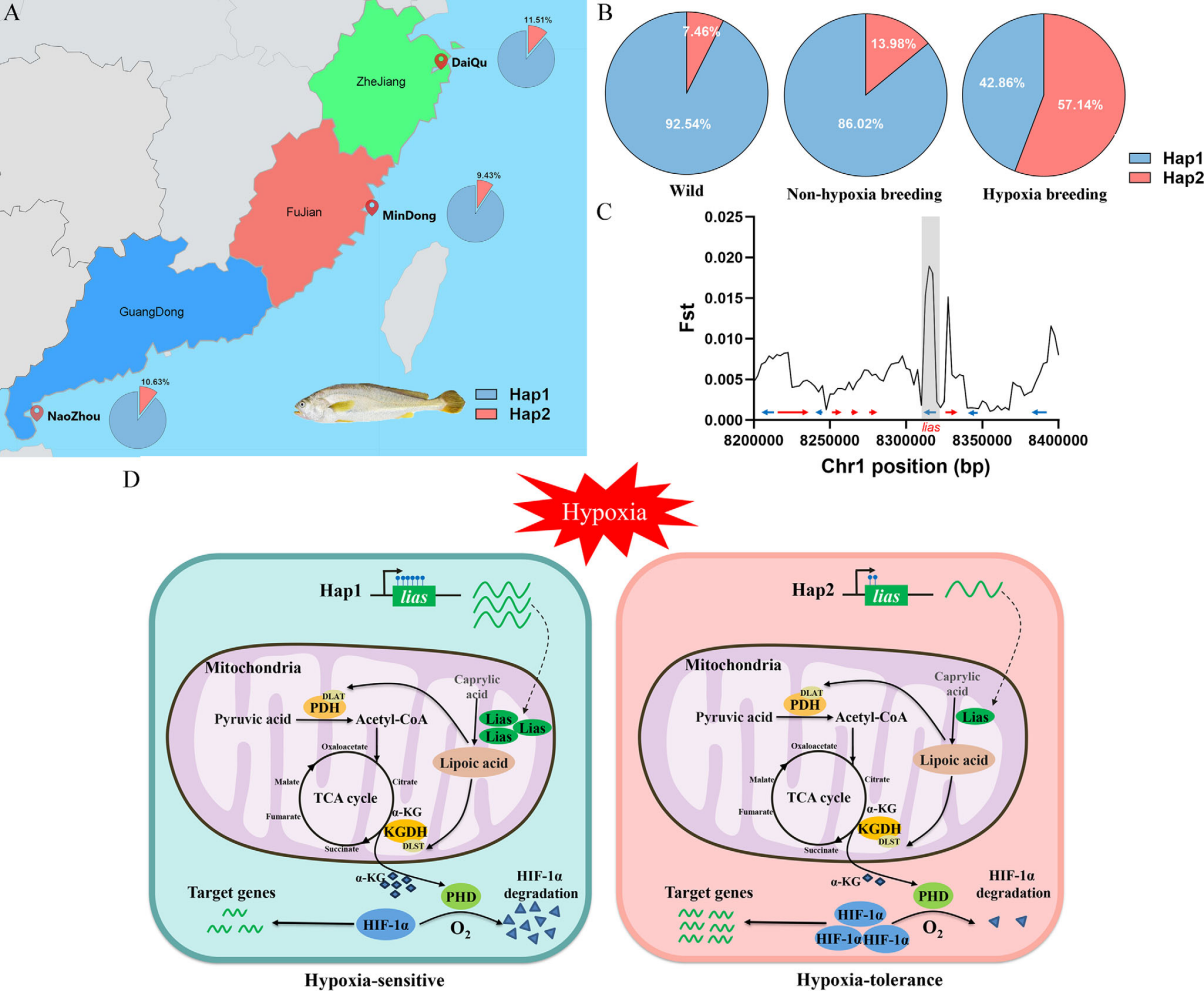
Geographic distribution and a working model of different *lias* haplotypes in *L. crocea* under hypoxia stress. (A) Distribution of *lias* haplotypes in three different populations. The pie chart in the figure shows the percentage of Hap2 in the DaiQu (*n* = 205), MinDong (*n* = 154), and NaoZhou (*n* = 47) populations. (B) *lias* different haplotype percentage statistics among different *L. crocea* types. (C) *F*st value detection in non‐hypoxia and hypoxia breeding *L. crocea* across the 200 kb genomic region containing the *lias* locus. Arrows indicate different genes and directions. (D) Proposed model of different *lias* haplotypes of *L. crocea* under hypoxia stress.

To illustrate the geographical distribution of the hypoxia‐tolerance allele, we investigated the *lias* haplotype in 406 *L. crocea* samples. The results revealed that the hypoxia tolerance allele *lias*‐Hap2 is widely distributed nationwide but at relatively low percentages (Figure 9A), specifically 11.51, 9.43, and 10.63% in the DaiQu, MinDong, and Nanzhou populations, respectively. However, this percentage is relatively lower in wild populations and tends to increase from an artificial breeding population (non‐hypoxia) to hypoxia breeding population (Figure 9B). *F*st analysis indicated a significant genetic differentiation trend between the non‐hypoxia and hypoxia breeding populations, suggesting that the different proportions of the *lias* haplotype may be associated with intensive artificial selection in *L. crocea* (Figure 9C). These results reveal the significant potential of *lias*‐Hap2 in *L. crocea* breeding programs to enhance hypoxia tolerance.

## 4 Discussion

Hypoxia stress occurs frequently in waterbodies; hence, hypoxia tolerance is a critical economic and complex developmental trait garnering considerable research attention. However, the mechanisms of hypoxia tolerance in fish are still not well understood. In this study, WGS and SNP‐chips data were integrated for GWAS and combined with transcriptome analysis to identify an important hypoxia tolerance candidate gene, *lias*. The gene function of *lias* and the regulation of HIF‐1α under hypoxia were then comprehensively explored, and the correlation between SNPs and hypoxia tolerance was preliminarily explained. This study may further elucidate the function of *lias* in regulating hypoxia tolerance in *L. crocea* and the potential role of related SNPs on this gene in genome breeding [39].

Initially, we explained how significant SNP affected the gene expression of *lias*. Generally, the functions of phenotype‐related SNPs varies according to their positions in the genome. SNPs located in protein‐coding regions can affect biological processes by directly regulating protein expression, while those in non‐coding regions have more complex regulatory mechanisms [40]. In GWAS, three SNPs associated with hypoxia tolerance in *L. crocea* were identified, all of which were located in the intronic region of the *lias* gene; among these, SNP LG1:8315503 was a CpG‐SNP. CpG‐SNP is an SNP mutation involving the addition or removal of the corresponding CpG site, such as A→G, C→T, and G→A transitions, which can alter the DNA methylation pattern in CpG sequences [41]. The association between CpG‐SNPs and certain phenotypes is being steadily explored [42]. However, majority of the functionally characterized CpG‐SNPs are located in the promoter or first exon regions and act by blocking the binding of relevant TFs. For example, the gene GDF5, which is associated with knee‐osteoarthritis, has a highly methylated CpG‐SNP site located in the promoter region, which inhibits the binding and thus promoter activity of the TFs SP1/SP3 [43]. In this experiment, an important TF, MAFK, was predicted in the region of the G allele at LG1_8315503. MAFK can interact with DNMT3L, an enzyme involved in *de novo* DNA methylation, by interacting with DNMT3A and DNMT3B [44]. Thus, the G allele can bind to the MAFK to promote the *de novo* DNA methylation of CGI2 and increase the methylation levels of the *lias* genes, wherein relatively lower methylation is associated with the A allele of the CpG‐SNP. This was also demonstrated using BSP, which showed that the methylation level of Hap1 was significantly higher than that of Hap2, and the DMR was mainly located in CGI2, a methylation island in the region where the CpG‐SNP is located. Although the negative correlation between DNA methylation and gene expression is well‐established [45], alternative mechanisms are still unclear, despite being reported in some studies. These studies have reported findings such as the methylation of the CpG island in intron 2 of the porcine gene G‐protein coupled receptor 120 promotes the transcription of its Mrna [46], high methylation of the CpG island in intron 1 of Early growth response protein 2 increases its gene expression [47], and hypermethylation of the CpG island from exon II to intron III on steroidogenic factor 1 (SF‐1) is highly positively correlated with SF‐1 expression in the stromal cells of endometriosis [48]. We speculate that the methylation of intronic regions as well as other gene body regions, may affect related cis‐regulatory elements, such as enhancers, thus promoting transcriptional activity and gene expression [49]. Further analysis of histone modifications has confirmed this. Region CGI2 is highly enriched in H3K4me3 and H3K27ac, and the enrichment level in Hap1 is higher than that in Hap2, indicating significant differences in their enhancer activity. Studies have shown that DNA methylation can directly prevent certain insulator proteins or transcriptional repressors from binding to DNA; conversely, when a region is highly methylated, these repressors are dislodged, thereby releasing previously silenced enhancers and allowing activator transcription factors or co‐factors to bind, thus enhancing transcriptional activity. For example, during early zebrafish development, active enhancers are typically highly methylated [50]. The imprinting control region immediately upstream of the *H19* gene contains multiple CTCF‐binding sites, and DNA methylation blocks the binding of CTCF, thereby increasing the interaction between the promoter of the paternal allele of *Igf2* and the enhancers, thus activating *Igf2* gene expression [51]. This conclusion were consistent with the observed high G‐allele‐dependent expression and methylation. In conclusion, we mechanistically characterized the role of CpG‐SNP LG1_8315503 in altering the transcriptional activity of *lias* through allele‐specific methylation.

Second, we functionally investigated how *lias* affects hypoxia tolerance. Lias is an important metabolic enzyme which is highly conserved in both prokaryotes and eukaryotes, and is predominantly localized in the mitochondria, where it primarily participates in LA synthesis in vivo [52]. In the present study, we have suggested HIF‐1α regulation as a novel effect of Lias. HIF‐1α is the core TF for hypoxia regulation, which regulates the adaptation of tissue cells to the hypoxia environment by activating a series of genes for energy metabolism, angiogenesis, redox homeostasis, and cell proliferation [53]. However, apart from the well‐established PHD, HIF‐1α is now known to be regulated by other genes or proteins [54]. The interference or overexpression of *lias* under hypoxia revealed that the protein accumulation of Hif‐1α and expressions of several downstream target genes of Hif‐1α were altered significantly, suggesting that *lias* downregulation might stabilize the protein expression of Hif‐1α. Under normoxia conditions, the half‐life of HIF‐1α is only a few minutes, as its proline residues are continuously hydroxylated by PHD and then degraded [55]. However, under hypoxic conditions, PHD activity is inhibited due to the decrease in oxygen concentration, resulting in the accumulation of excess HIF‐1α due to inhibition of the degradation process. Since PHD is a member of the α‐KG‐dependent dioxygenase family, its activity is regulated by both oxygen concentration and α‐KG, and the activity of PHD under hypoxia also depends on the α‐KG concentration [56]. Therefore, we suggest that the downregulation of *lias* increased HIF‐1α protein expression presumably via the metabolic inhibition of PHD activity, which was confirmed by the changes of hydroxylated HIF‐1α and α‐KG content following interference or over‐expression. Since α‐KG is a key intermediate metabolite in the TCA cycle, *lias* may potentially regulate the activities of related enzymes in the TCA cycle, which leads to a decrease in the α‐KG content. However, studies suggest that α‐KG may influence prolyl hydroxylase activity by converting into L‐2‐HG [54]. Metabolomics results revealed no significant difference in intracellular 2‐HG levels between the si_*lias* and si_NC groups. Interference or overexpression of 2HGDH (a mitochondrial protein catalyzing L‐2‐HG oxidation to α‐KG) in human pulmonary arterial endothelial cells (PAECs) and pulmonary arterial smooth muscle cells (PASMCs) did not alter HIF‑1α target gene expression (GLUT1, LDHA), suggesting HIF‑1α stabilization occurs independently of direct 2‑HG‑mediated PHD inhibition [57]. Related studies also emphasize the importance of glutamine catabolism in malignant tumor cells under hypoxia. Based on this, we think that the primary carbon source for L‐2‐HG production in PAECs is glycolysis, whereas in HeLa and 293T cells, the primary carbon source may be glutamine catabolism, leading to differing experimental outcomes. Therefore, this mechanism may differ between primary cultured cells (such as PAECs, PASMC, and LYCF cells used in this experiment) and immortalized cell lines (293T and HepG2). Additionally, if this mechanism depends on L‐2‐HG, the addition of α‐KG should result in an upward trend in HIF1‐α protein levels. However, our experiments yielded the exact opposite outcome, consistent with similar results reported in other studies. Zhao et al. identified base mutations in isocitrate dehydrogenase 1 (IDH1) that suppressed IDH1 catalytic activity in primary glial cells, thereby reducing α‐KG production and increasing HIF‐1α [58]. Paredes et al. demonstrated a significant reduction in HIF‐1α protein abundance upon α‐KG addition in HASMC cells [59]. Hou et al. observed a 50% downregulation of HIF‐1α protein levels 60 min after Octyl‐2KG (an α‐KG derivative) treatment in MDA‐MB‐231 cells [60]. Therefore, we speculate that the effects of α‐KG on HIF‐1α may involve entirely distinct mechanisms across different cell types, which require further investigation.

LA, which covalently attaches to lysine residues of proteins via amide bonds to form lipoylated proteins, is a highly conserved post‐translational modification of proteins from bacteria to mammals [61]. To date, only four lipoylation‐modified proteins, PDH complex, α‐KGDH complex, GCSH, and BCKDH, have been identified in organisms [59, 62]. The present study findings indicate that the interference or overexpression of *lias* affected the enzymatic activities of PDH and α‐KGDH complex. The PDH complex consists of three components: PDH, DLAT, and dihydrolipoic amide dehydrogenase, and LA is the coenzyme for DLAT [63]. Moreover, α‐KGDH is a complex enzyme, and the cofactor of its E2 component, DLST, is also LA [64]. The activities of the two lipoylation proteins, DLAT and DLST, were significantly altered after interfering with *lias*, implying that *lias* regulates enzyme activities by modulating the lipoylation of DLAT and DLST, which in turn decreases the rate of pyruvate metabolism and TCA cycle, along with decreased α‐KG accumulation, which inhibits PHD activity as well as promotes the accumulati on of HIF‐1α. This phenomenon has been demonstrated previously wherein patients with inborn defects in LA anabolism exhibit lower activities of the PDH complex and lipoylated proteins than those in normal individuals [54, 62]. Two other lipoylation proteins (GCSH and BCKDH) are mainly associated with glycine metabolism and branched‐chain amino acid metabolism. Glycine metabolism may influence one‐carbon metabolism and redox balance, while changes in BCKDH activity may affect the utilization of energy substrates. These effects may synergistically regulate the hypoxic response through changes in TCA cycle flux mediated by DLAT/DLST, however, the specific mechanisms by which they influence HIF‐1α remain unclear and require further investigation.

## 5 Conclusion

We elucidated the functional mechanism of the remarkable SNPs found on *lias* during hypoxia tolerance in *L. crocea*. Mechanistically, the CpG‐SNP LG1_8315503 alters the enhancer activity and subsequent transcriptional activity of the *lias* through allele‐specific methylation. Functionally, *lias* regulates the α‐KG content, which in turn negatively regulates the protein level of HIF‐1α through PHD, resulting in better tolerance to the hypoxia environment (Figure 9D). This study identified *lias* as a previously unrecognized gene regulating hypoxia tolerance in *L. crocea*. Further studies of upstream regulatory genes may help identify the polymorphisms critical for *lias* transcription, thus better elucidating the regulatory network. Our study provides a new research perspective for the theory of fish hypoxia tolerance. It identifies accurate targets for the development of hypoxia tolerance molecular markers and gene editing and breeding [13, 39, 65], which has important theoretical significance and potential application value.

## Author Contributions

**Yibo Zhang**: conceptualization, software, methodology, supervision, writing – original draft, writing – review and editing, resources. **Ran Meng**: validation, conceptualization. **Junquan Zhu**: writing – review and editing, supervision, resources, funding acquisition, project administration. **Songpeng Jia**: validation, formal analysis. **Jie Ding**: conceptualization, methodology, software, data curation, investigation, validation, formal analysis, visualization, writing – original draft, writing – review and editing. **Weiliang Shen**: funding acquisition, project administration, resources, supervision. **Xuelei Wang**: validation, methodology. **Xiongfei Wu**: project administration, funding acquisition, supervision, resources.

## Funding

This research was funded by the National Key Research and Development Program of China (NO. 2022YFD2401003), the China Agriculture Research System of MOF and MARA (NO.CARS‐47), the Postdoctoral Fellowship Program of CPSF (NO. GZC20252052), the China Postdoctoral Science Foundation (NO. 2024M761533), the Science and Technology Innovation 2025 Major Special Project of Ningbo City (No. 2021Z002), the Zhejiang Science and Technology Major Program on Agricultural New Variety Breeding (NO. 2021C02069‐1‐2), and the K.C. Wong Magna Fund in Ningbo University.

## Conflicts of Interest

The authors declare no conflicts of interest.

## Supporting information




**Supporting File 1**: advs76310‐sup‐0001‐SuppMat.pdf.

**Supporting File 2**: advs76310‐sup‐0002‐TablesS1‐S15.xlsx.

## Data Availability

The data that support the findings of this study are available from the corresponding author upon reasonable request.
