# Supplementary material for: Integrative Omics Analysis Reveals the Regulation of Hypoxia Tolerance in Large Yellow Croaker (Larimichthys crocea) via the Lipoic Acid Synthase (lias) Gene
Source: Adv Sci (Weinh). 2026 Jun 26;13(53):e76310. doi: 10.1002/advs.76310 (PMC13336880; doi:10.1002/advs.76310)
Supplement: Supplementary file 1 [file ADVS-13-e76310-s002.pdf]

Supplementary information for the manuscript entitled

"Integrative omics analysis reveals the regulation of hypoxia tolerance in large yellow croaker (*Larimichthys crocea*) via the lipoic acid synthase (*lias*) gene"

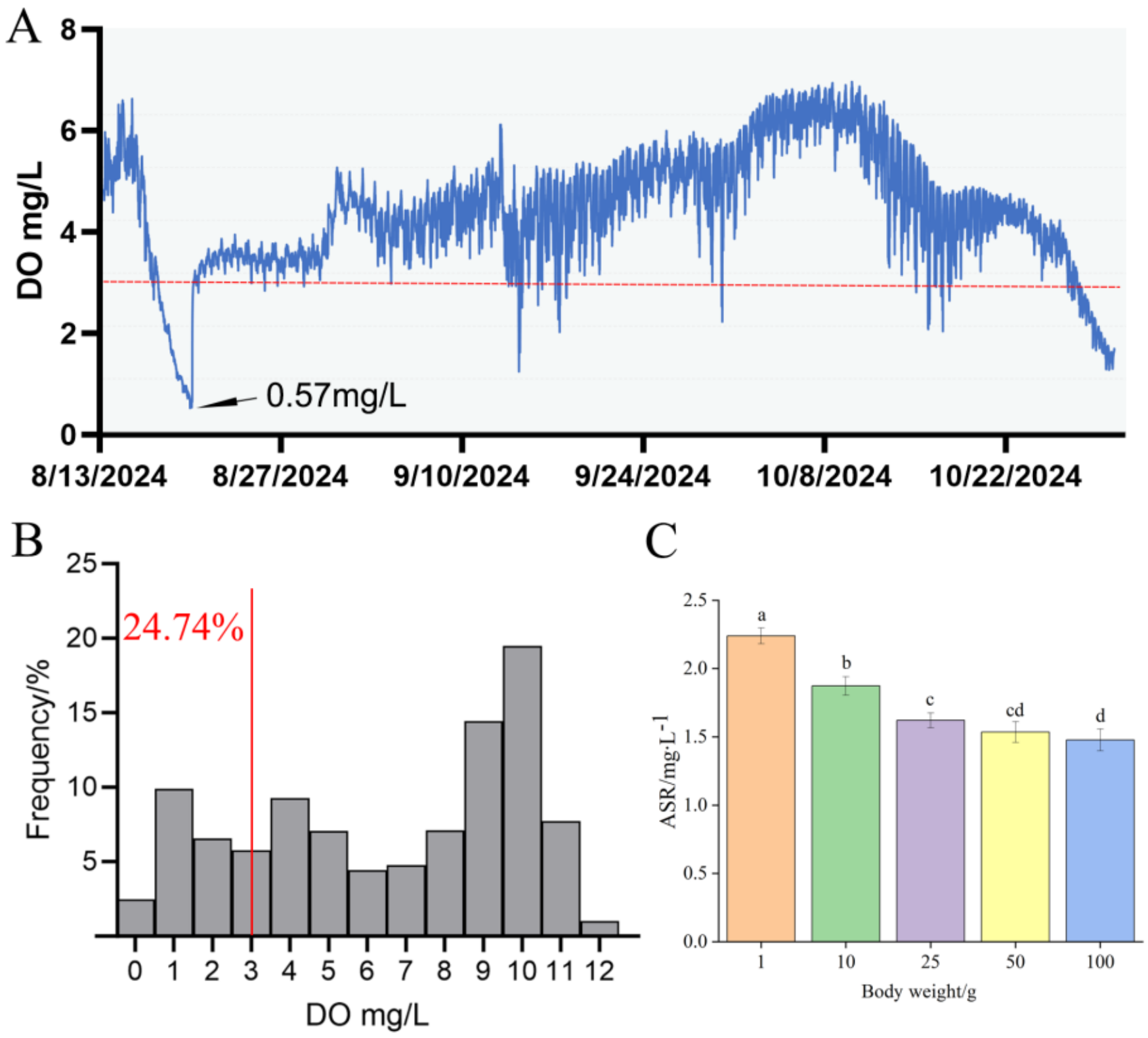

Fig. S1. Dissolved oxygen (DO) monitoring and floating head behavior analysis of *Larimichthys crocea* sea-cages. (A) Changes of DO levels in a certain *L. crocea* sea-cage from August to October 2024. The detection frequency is once every 10 minutes. The red dotted line represents the DO concentration (3.0 mg/L) required for the normal growth of *L. crocea*. (B) Frequency distribution histogram of daily average DO value in a certain *L. crocea* sea-cage from August to October 2024. (C) The DO of floating head of *L. crocea* with different body weights. Different letters indicate statistically significant differences at $P < 0.05$.

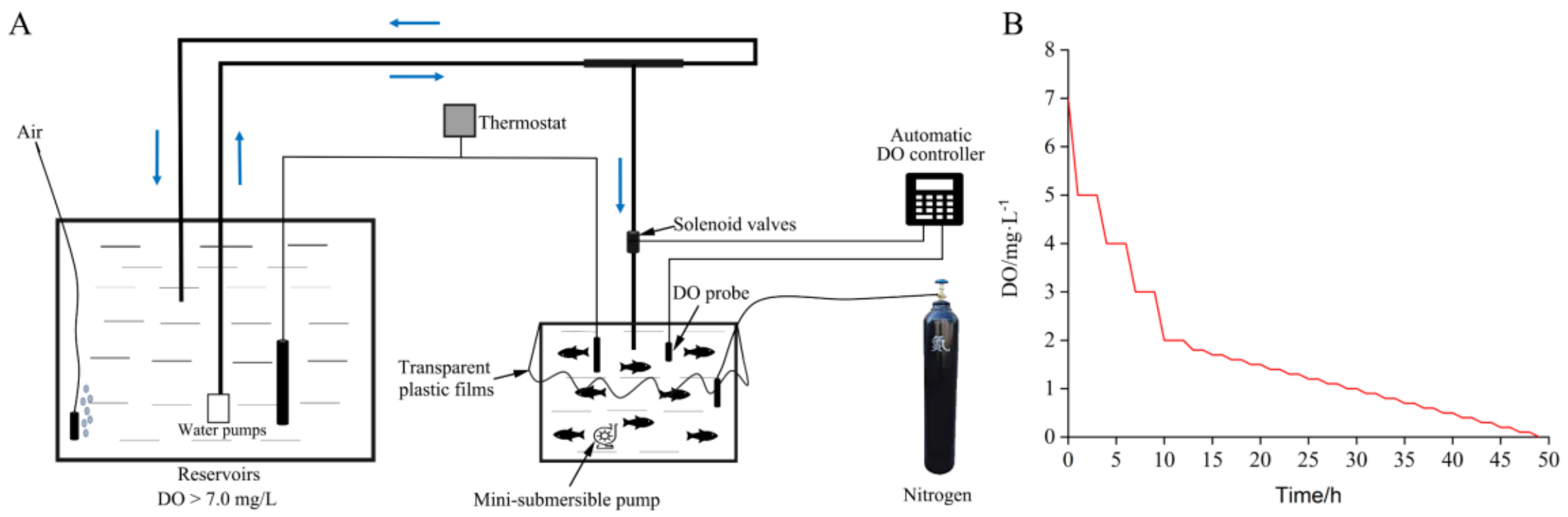

Fig. S2. Hypoxia experiments of *Larimichthys crocea*. (A) A brief diagram of the hypoxia stress experiment. (B) Curve of DO changes for *L. crocea* under hypoxia stress.

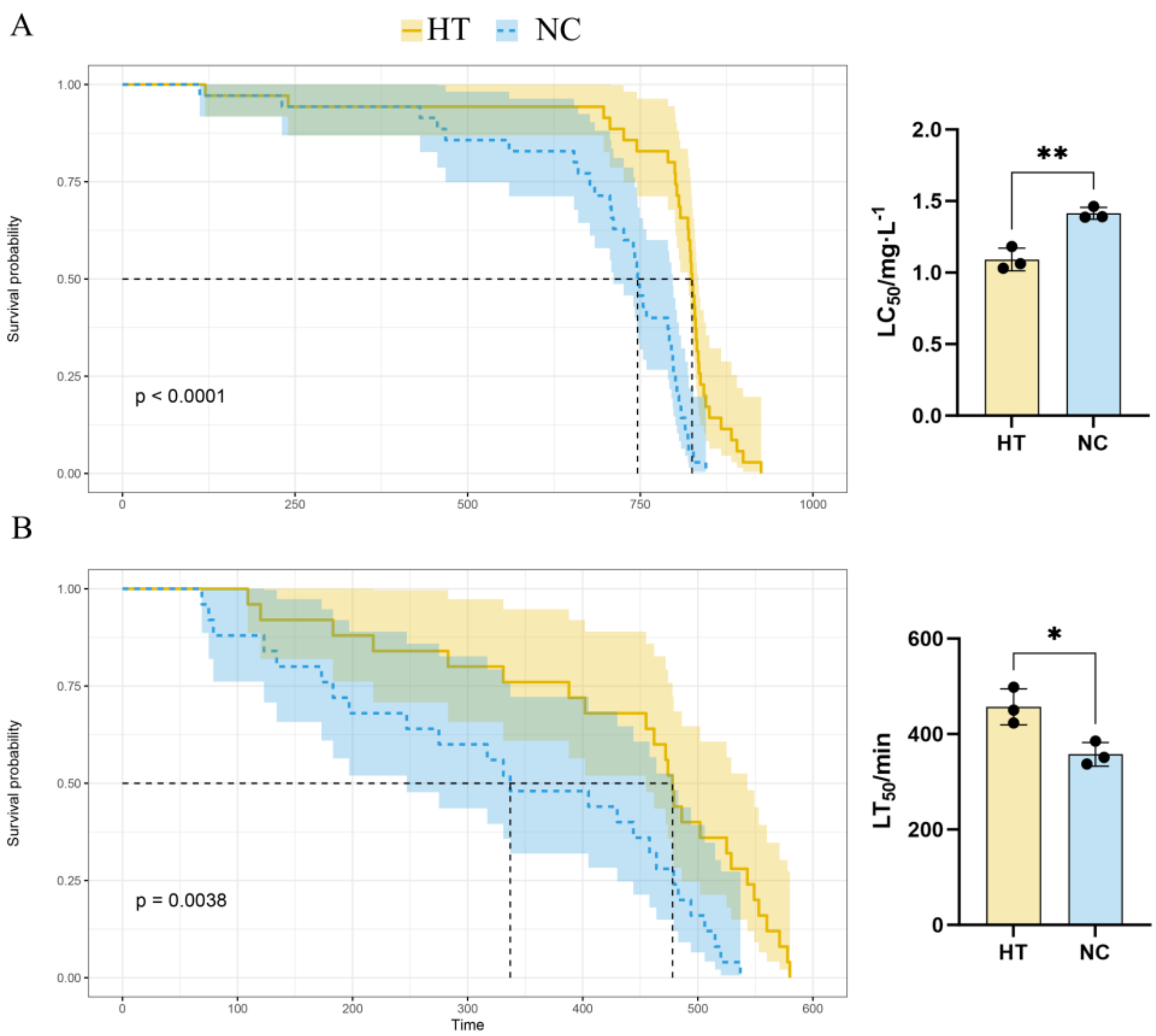

Fig. S3. Hypoxia tolerance evaluation tests for the hypoxia-tolerant group (HT, T) and the control group (NC, N). (A) The survival analysis of HT and NC under gradient oxygen reduction. (B) The survival analysis of HT and NC under acute hypoxia stress (DO = 1.5 mg/L).

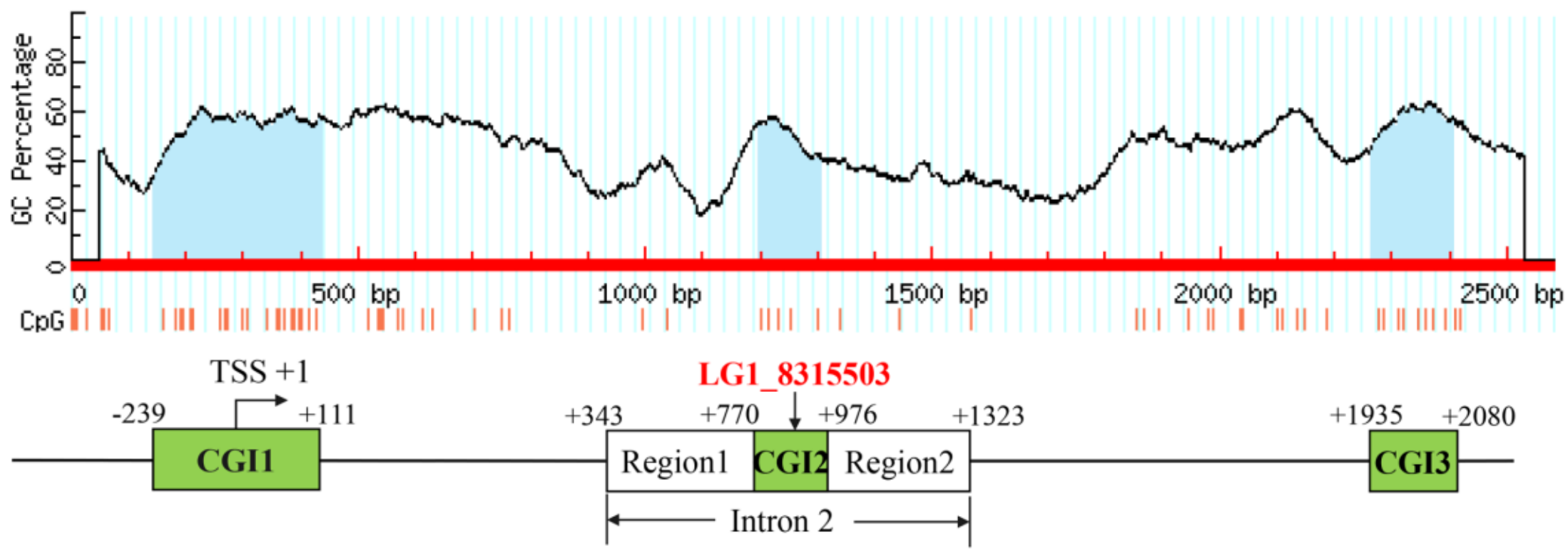

Fig. S4. Three putative CpG islands (CGI) in the *lias* genomic sequence predicted by Methyl-primer software (https://www.methprimer.com/). Criteria used: Island size > 100 bp, GC percent > 50.0, Observed/expected CpG ratio > 0.6. Location of CGI1, CGI2, and CGI3 in chromosome 1: 8216649-8316299, 8315640-8315434, and 8314475-8314330.

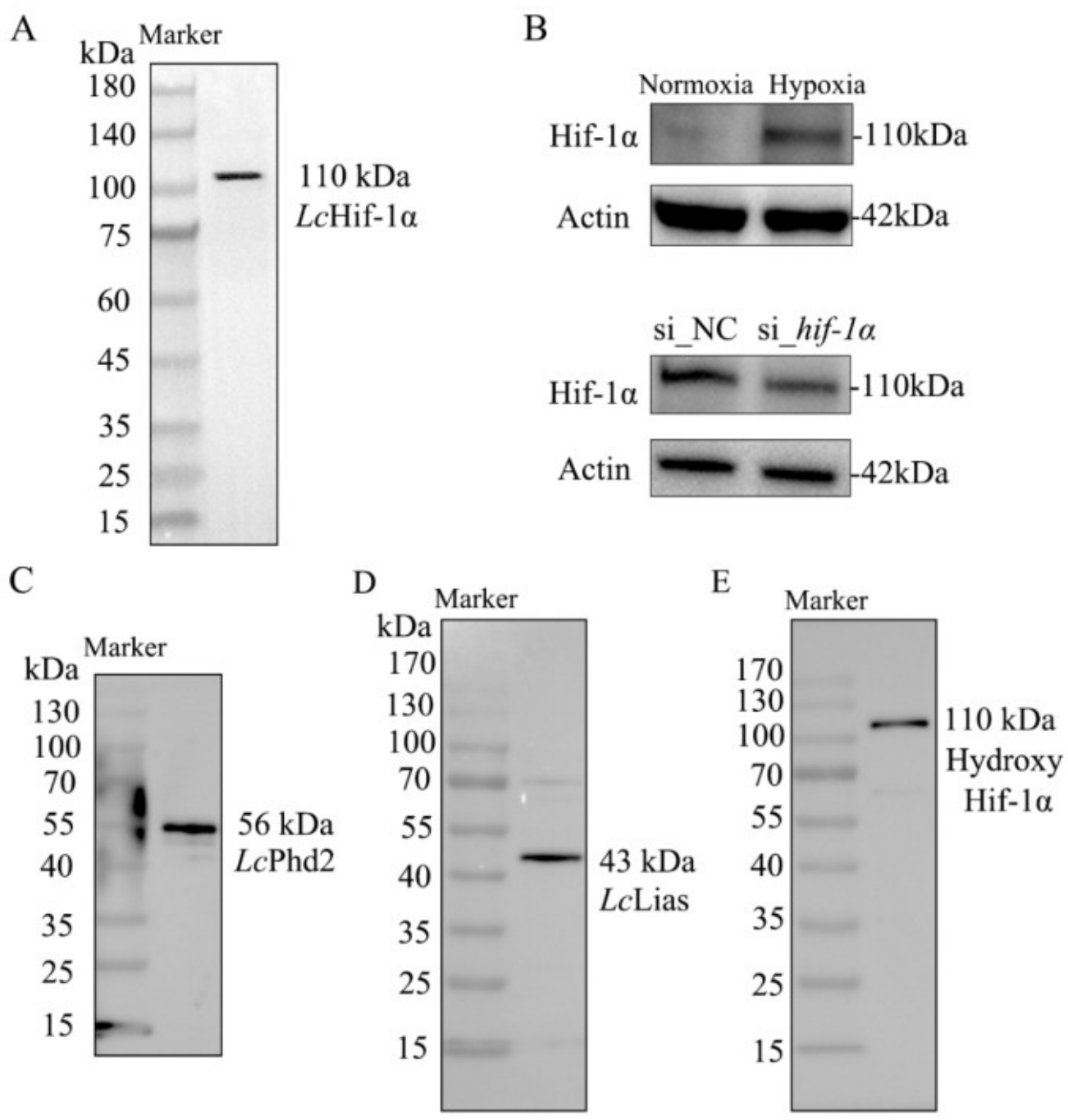

Fig. S5. Verification of self-made antibody and commercial antibody. (A) Western blot validation of the specificity of the *Lc*Hif-1α antibody. (B) Changes in Hif1-α protein expression in LYCF cells under hypoxia and si_*hif-1α*. (C) Western blot validation of the specificity of the *Lc*Phd2 antibody. (D) Western blot validation of the specificity of the *Lc*Lias antibody. (E) Western blot validation of the specificity of the Hydroxy-Hif-1α (Pro564) antibody.

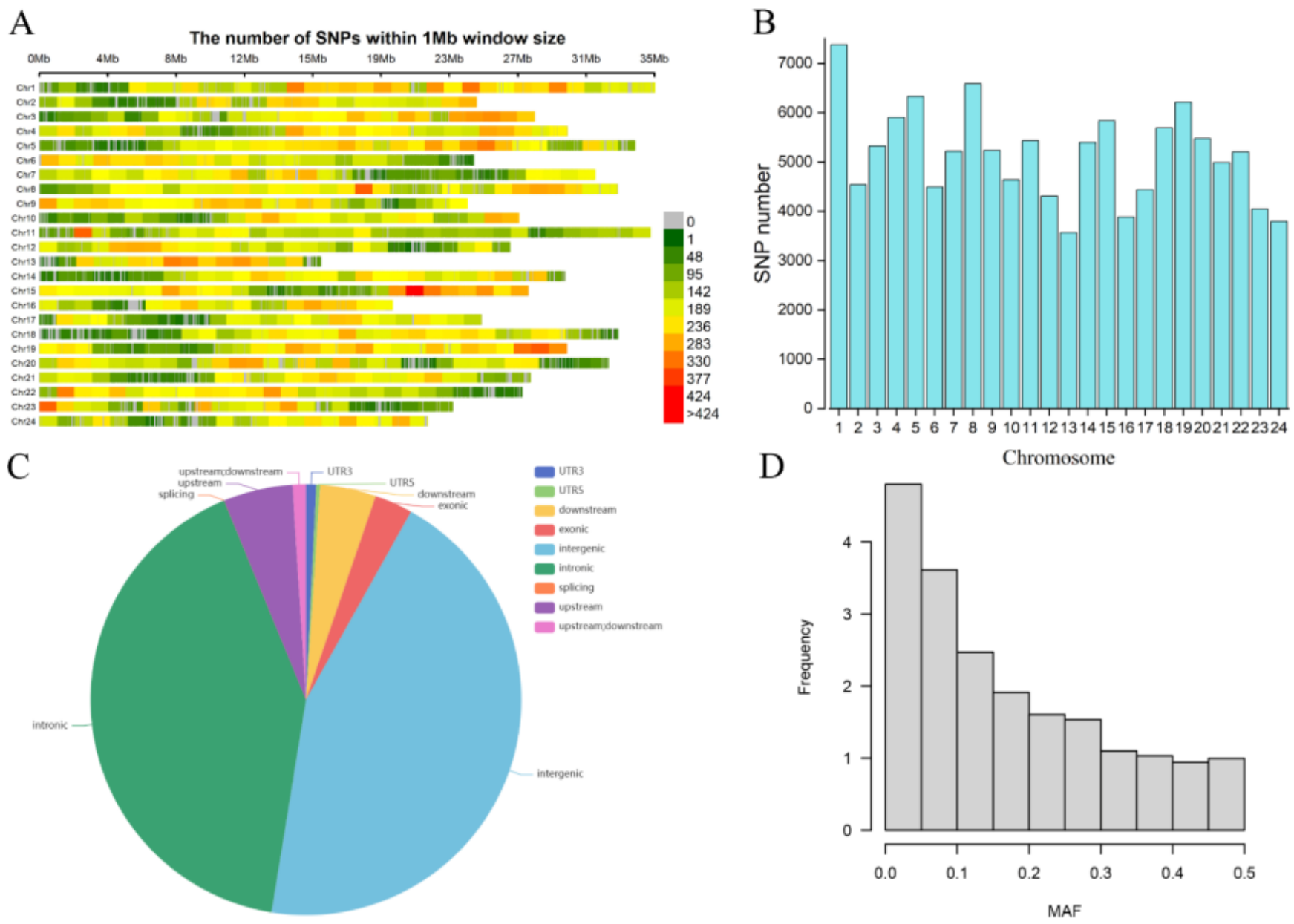

Fig. S6. Statistical data of genotyping based on SNP_chip. (A) Density distribution map and (B) columnar distribution map of SNP from SNP chips in each chromosome. (C) SNP annotation statistics. (D) Histogram of the MAF distribution of SNPs.

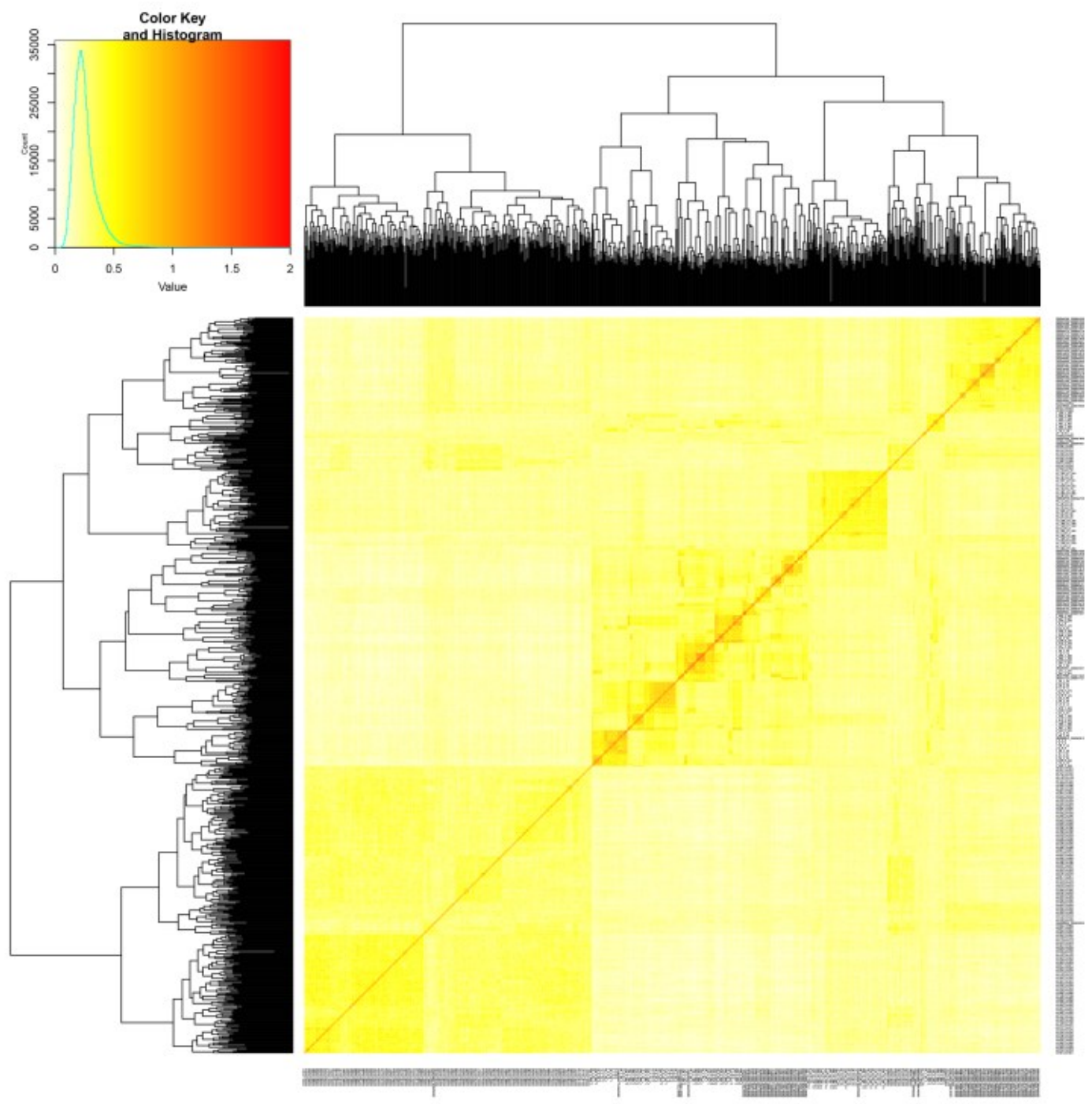

Fig. S7. A heatmap depicting genetic kinships among the individuals of *Larimichthys crocea* based on SNP_chip data. The green curve of genetic relatedness indicates that most of the relatedness values are distributed around a value of 0 (the yellow region).

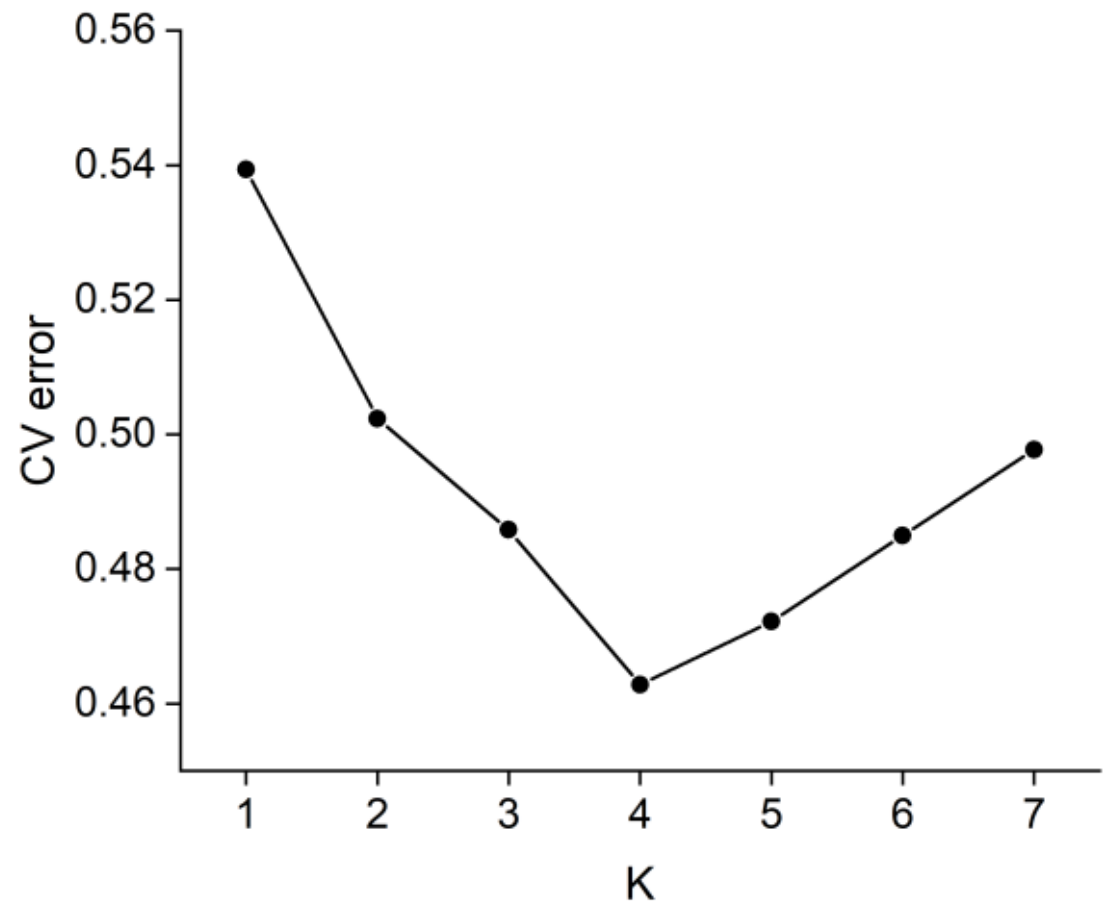

Fig. S8. The change of cross validation error at different K-value in population structure analysis.

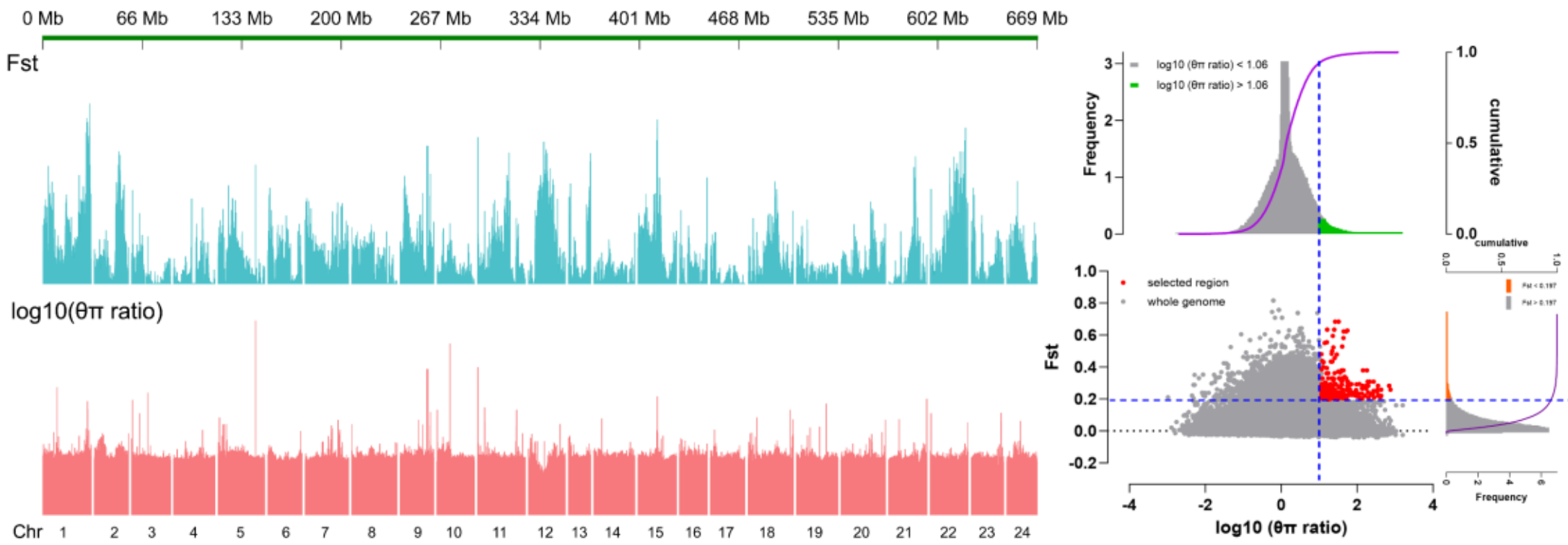

Fig. S9. Genetic differentiation index (*F*st) and Nucleotide diversity (θπ) analysis for hypoxia-selected populations and unselected populations. The significance threshold of the selection signature was arbitrarily set to the top 5% percentile outliers for each test. The scatter plot on the right shows the selected regions associated with hypoxia tolerance of *Larimichthys crocea* identified through joint *Fst* and θπ ratios.

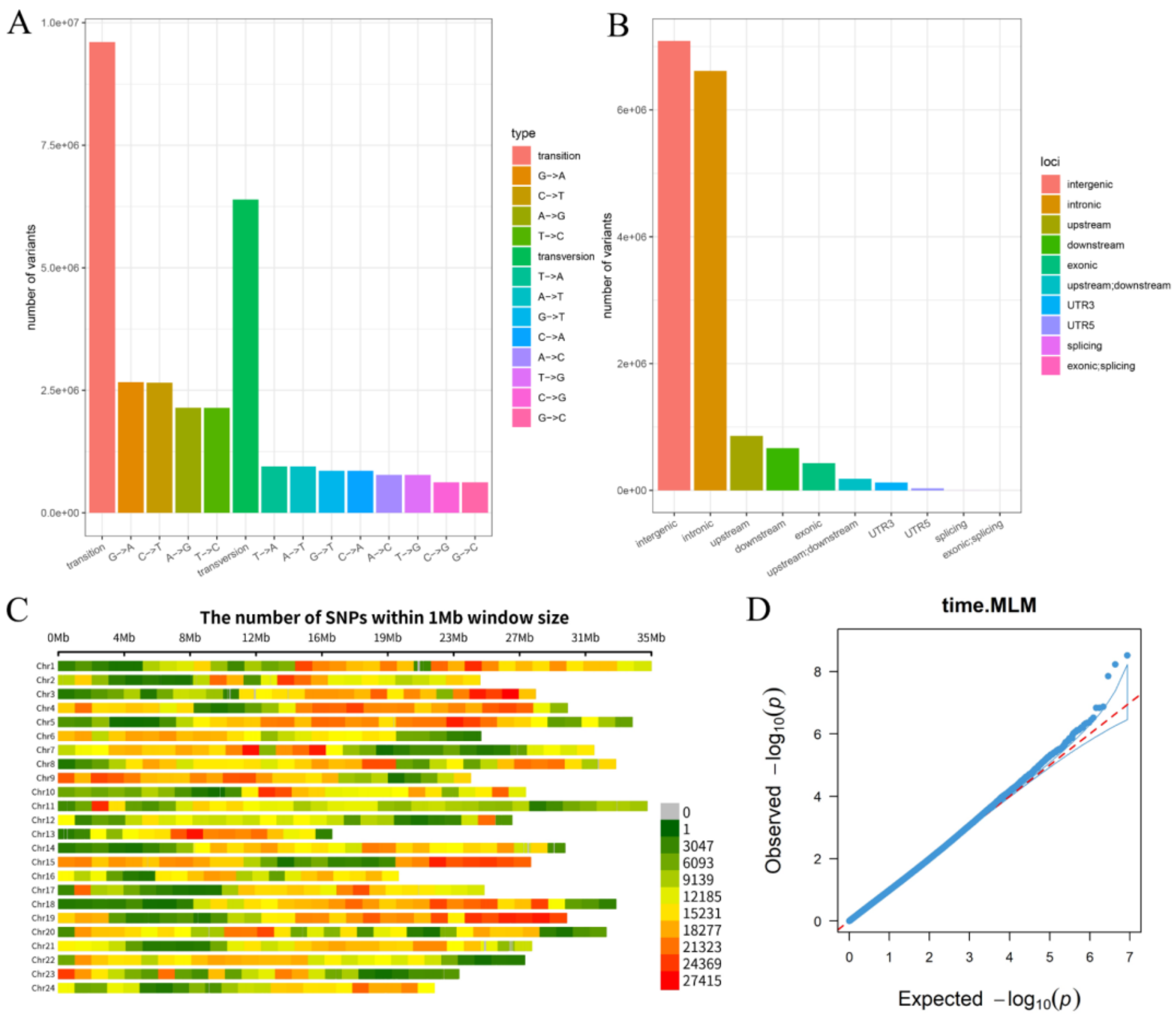

Fig. S10. Statistical data of genotyping based on WGS data. (A) Statistical chart of SNP types. (B) Statistical chart of SNP position annotation. (C) Density distribution map; (D) Q-Q plot of GWAS for hypoxia tolerance of *L. crocea* with WGS data.

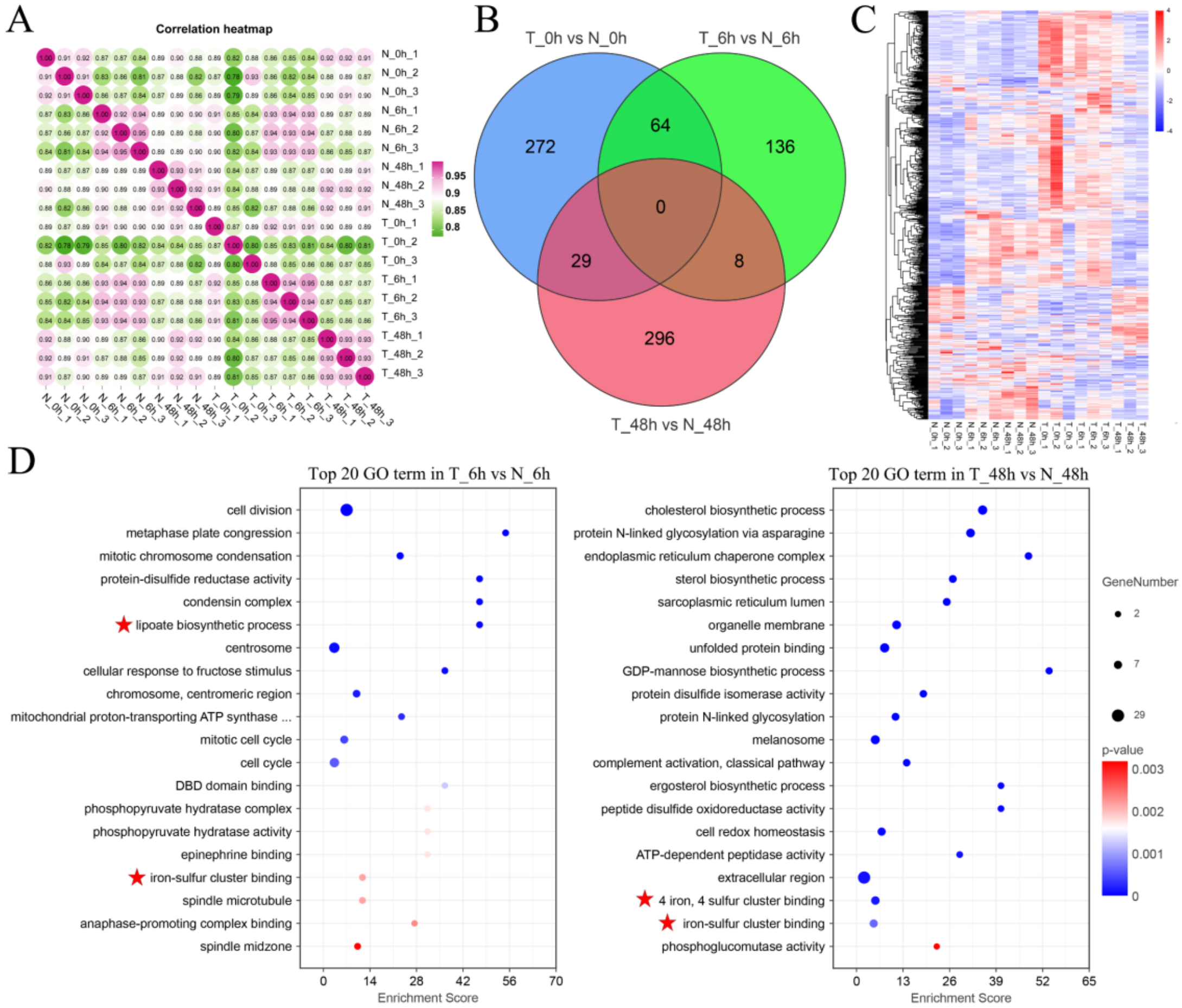

Fig. S11. Transcriptome analysis under hypoxia stress in hypoxia-tolerant (T) and control (N) groups of *Larimichthys crocea*. (A) Correlation heat map between different transcriptome samples. (B) Venn diagram of DEGs in three groups: T_0h vs N_0h, T_6h vs N_6h, and T _ 48h vs N _ 48h. (C) Heat maps of DEGs between T and N group. (D) Go enrichment analysis of DEGs between T and N group (Top 20 GO term).

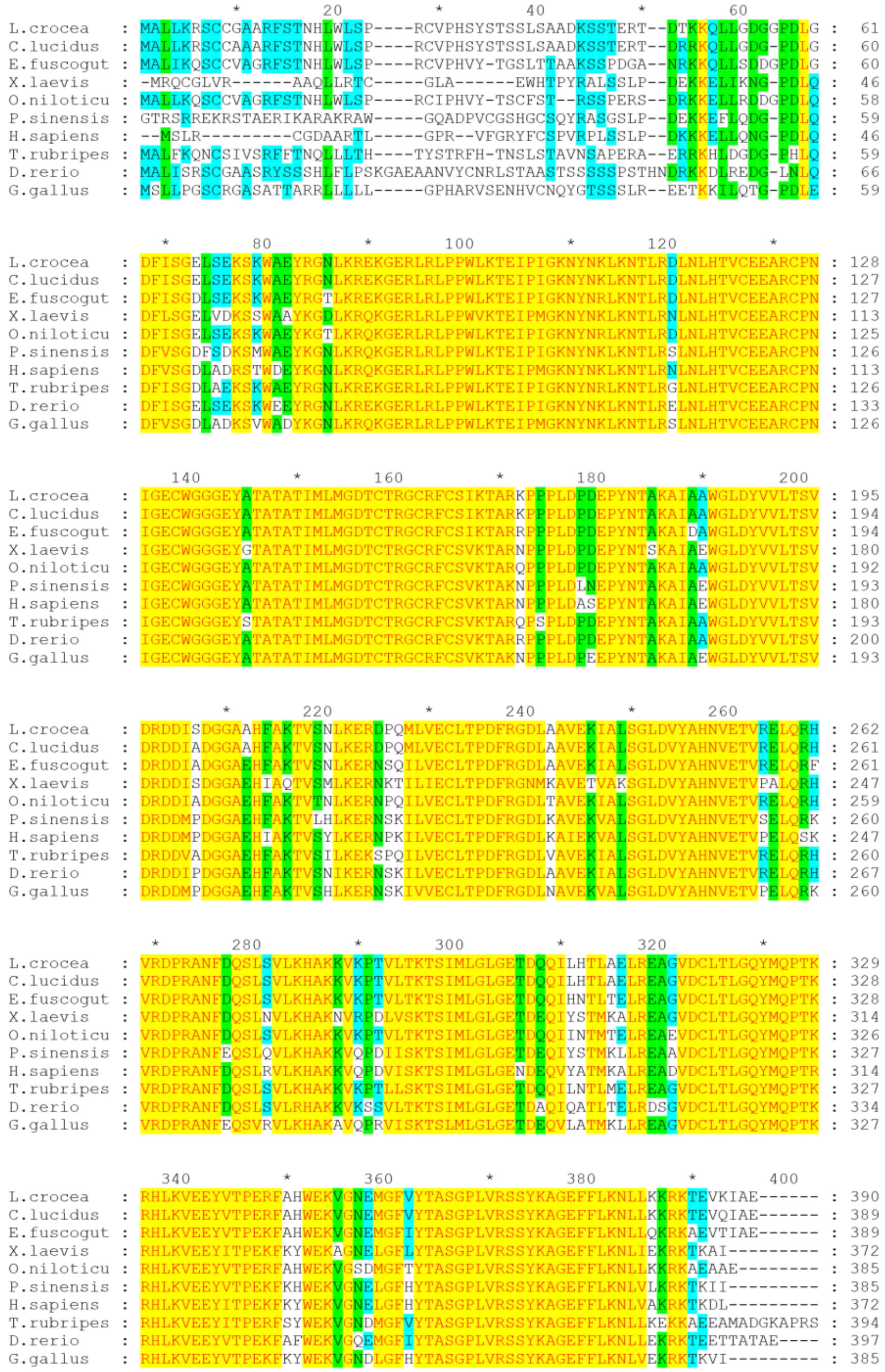

Fig. S12. **Multiple sequence alignment of Lias homologous proteins.** The same amino acids are high lighted in yellow, the bluish green regions show more than 50% similarity, and the green region shave a lower similarity. The consensus positions of *Larimichthys crocea* Lias with Lias in *Collichthys lucidus*, *Epinephelus_fuscoguttatus*, *Xenopus_laevis*, *Oreochromis_niloticus*, *Pelodiscus_sinensis*, *Homo_sapiens*, *Takifugu_rubripes*, *Danio_rerio, and Gallus_gallus* was 93.19, 83.83%, 83.24%, 82.98%, 78.42%, 77.89%, 74.48%, 73.20%, and 70.70%, respectively.

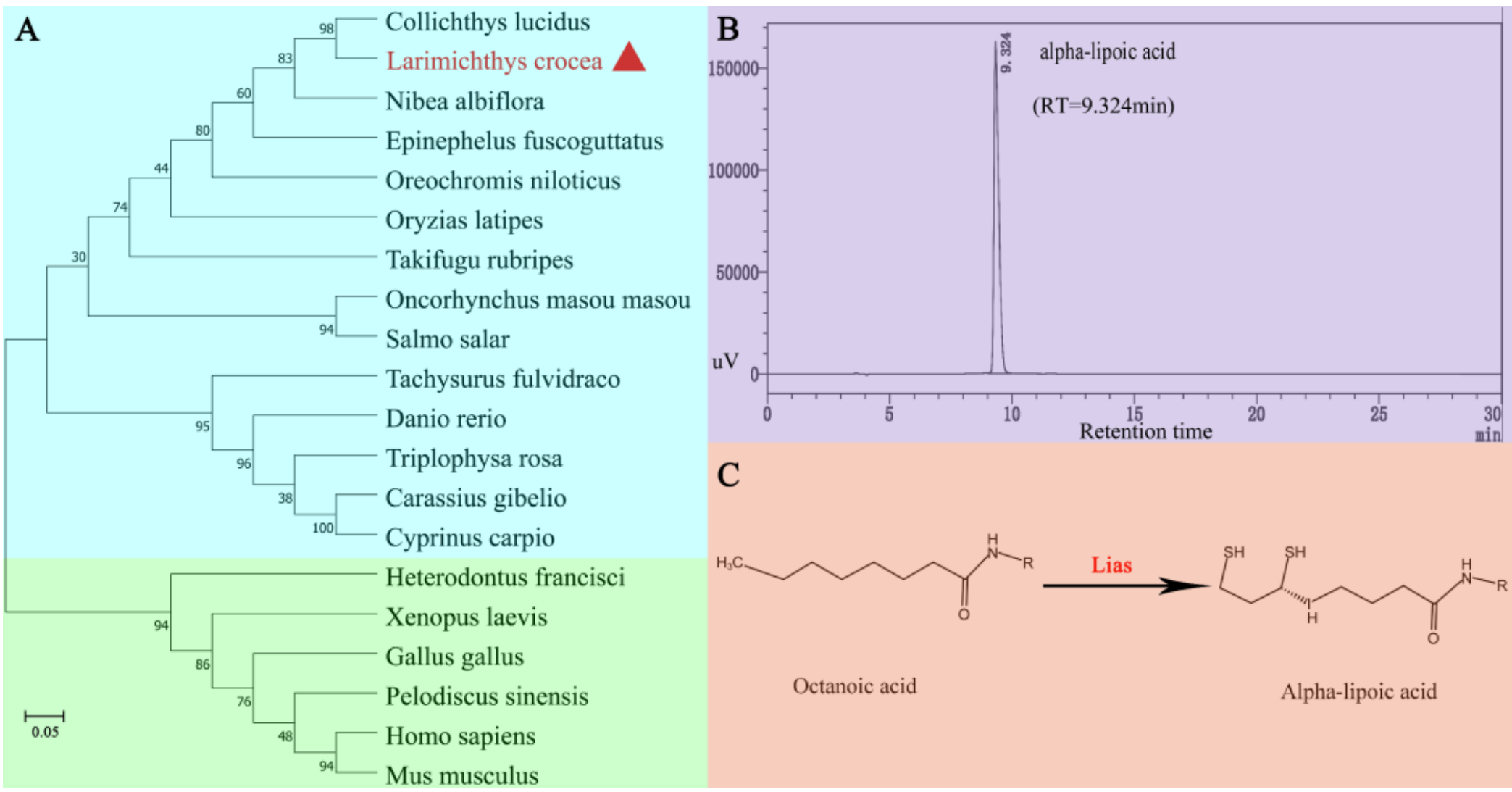

Fig. S13. **Phylogenetic and functional analysis of Lias.** (A) Phylogenetic tree based on the amino acid sequence of Lias proteins. The Mega5.1 software was used to construct the NJ phylogenetic tree. The *Larimichthys crocea* is marked in red font. (B) Gas chromatography of the product catalyzed by Lias. (C) Proposed pathway of alpha-lipoic acid biosynthesis in *L. crocea*.

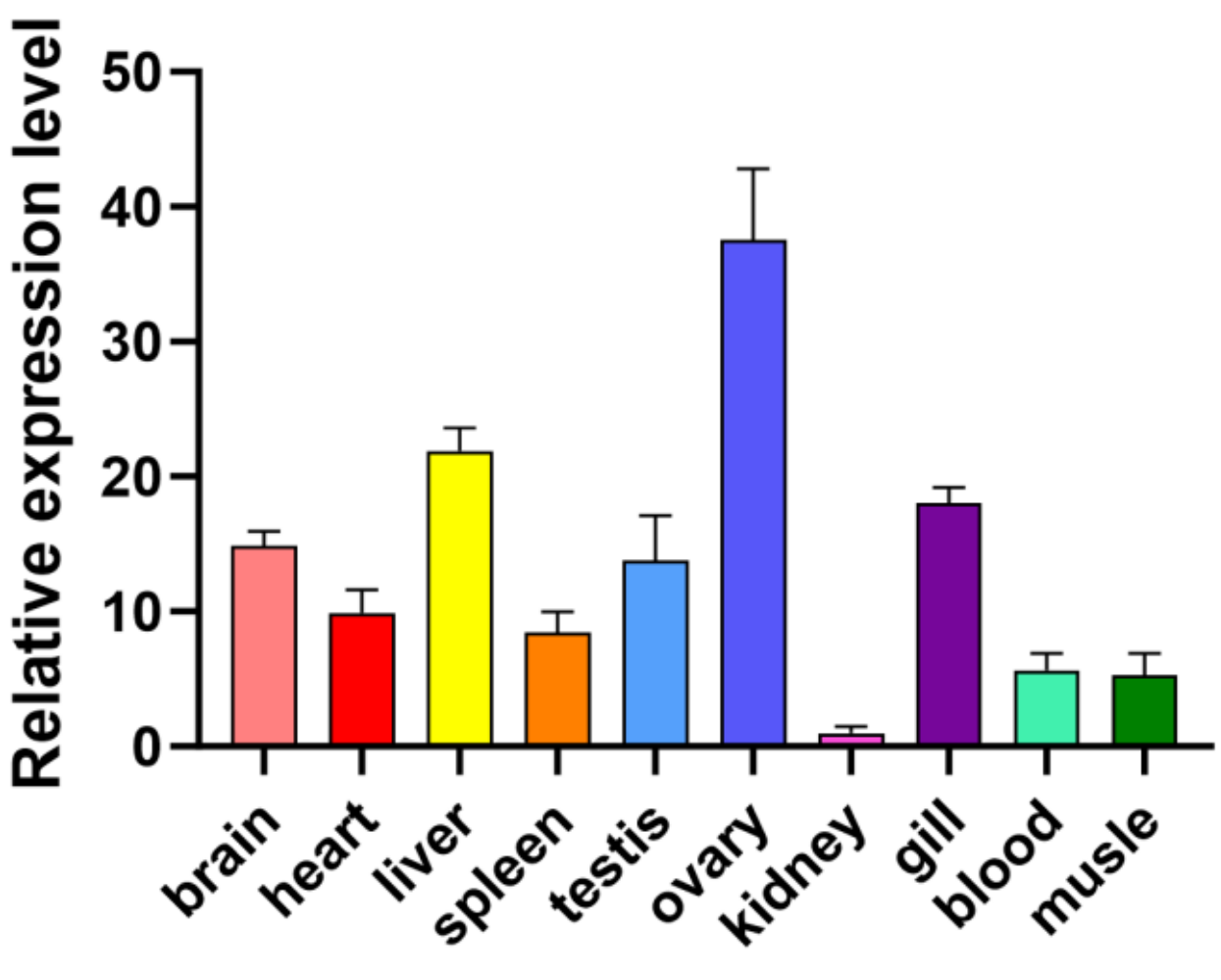

Fig. S14. Expression of *lias* mRNA in various tissues of *Larimichthys crocea.*

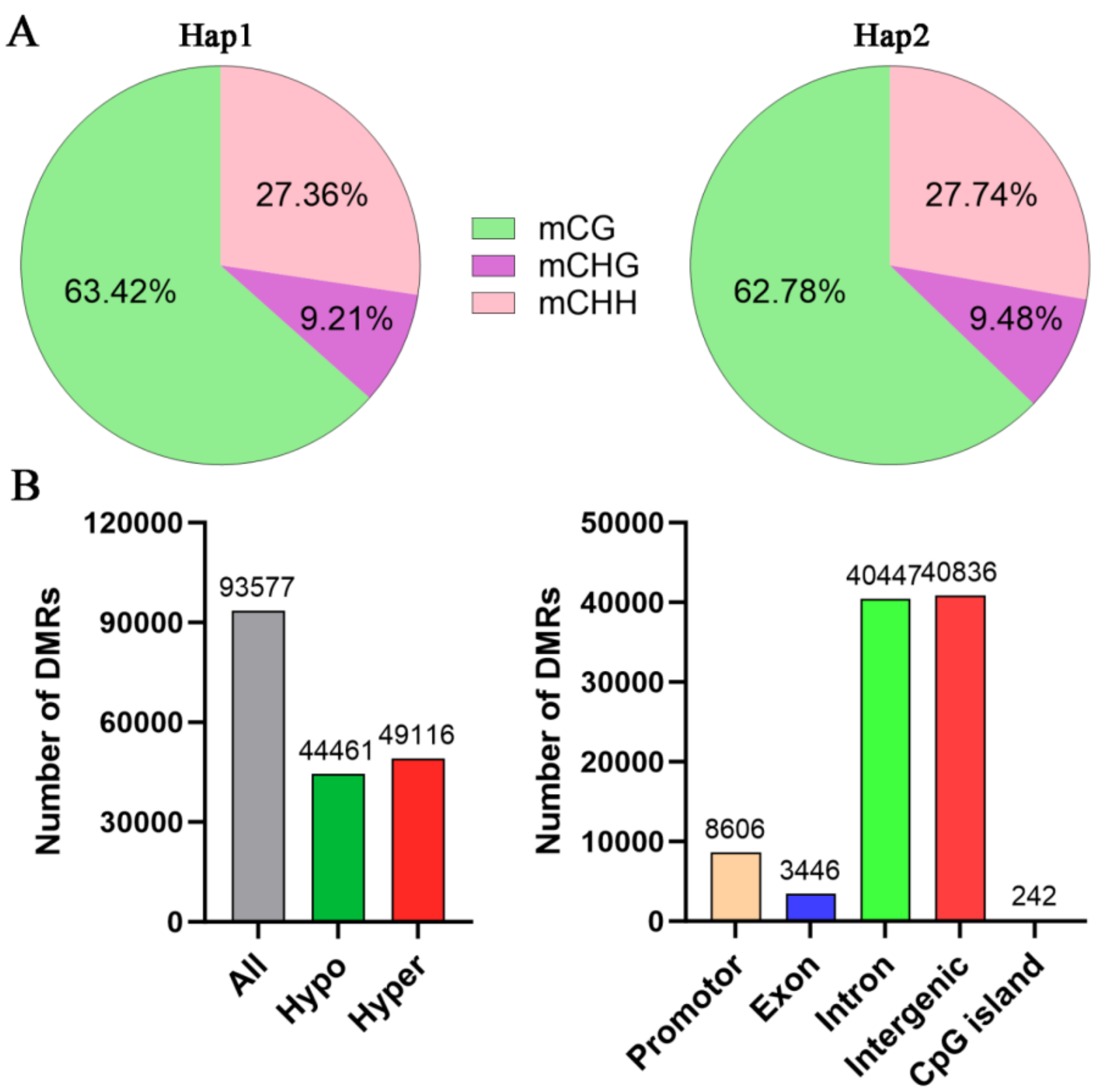

Fig. S15. Distribution and characterization of mCG, mCHG and mCHH in the two haplotypes and identification of differentially methylated regions (DMR) between the two haplotypes. (A) Percentage distribution of different types of methylated C bases. The whole circle represents all mC (100%) of the whole genome, the green part is mCG, the pink part is mCHG, and the purple part is mCHH. (B) Number of DMRs (hyper- and hypo-methylated) and their distribution in different genomic elements.

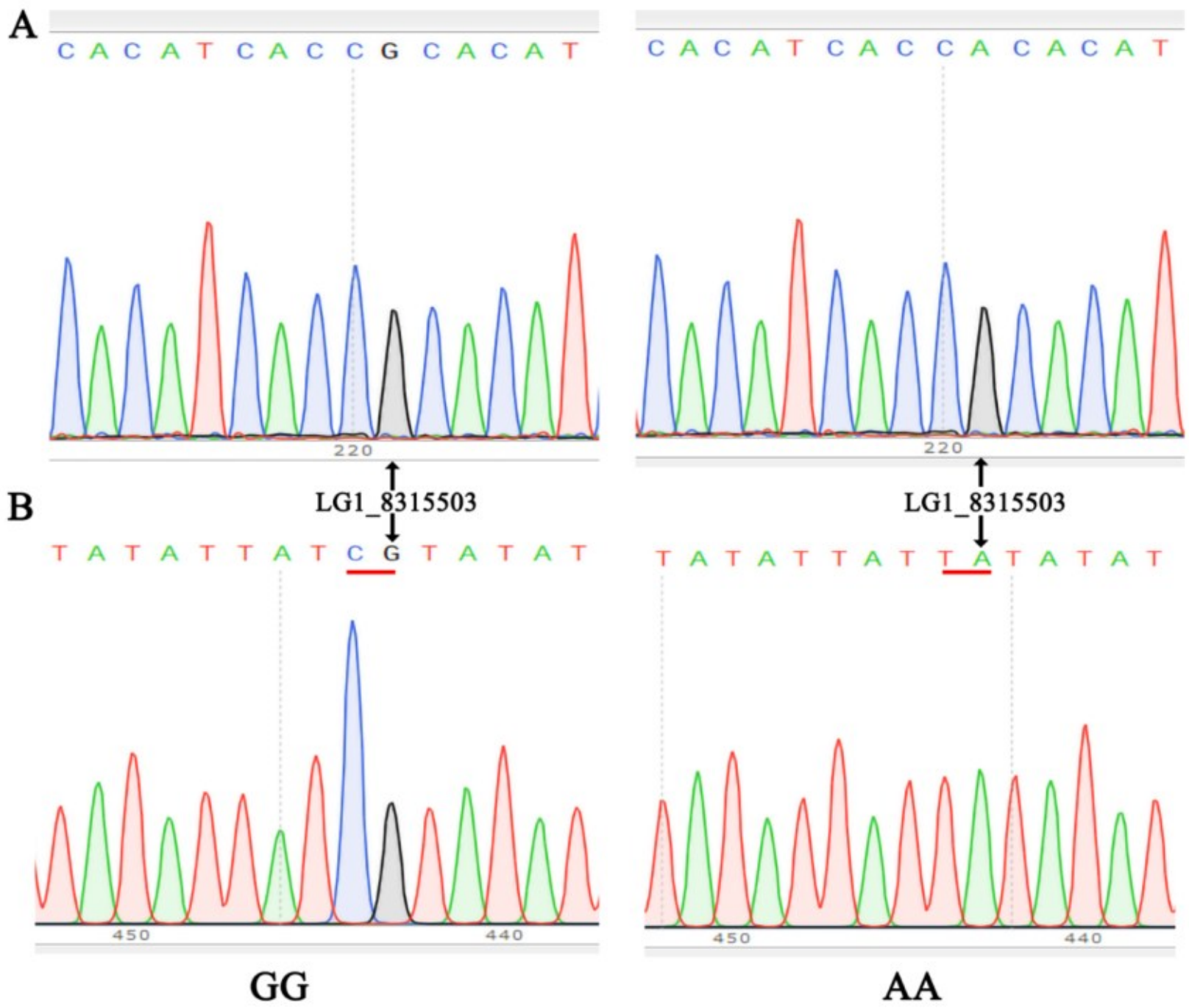

Fig. S16. Partial sequences of intron 2 of the *lias* gene in DNA samples from G/G and A/A individuals. (A) and (B) show genomic DNA and bisulfite-treated DNA sequencing of the LG1_8315503 region, respectively. The major G allele forms a CpG site, but it does not exist in the A allele. After bisulfite treatment, the CpG site dependent on G allele was completely methylated, but cytosine (C) was converted into thymine (T) in AA genotype.

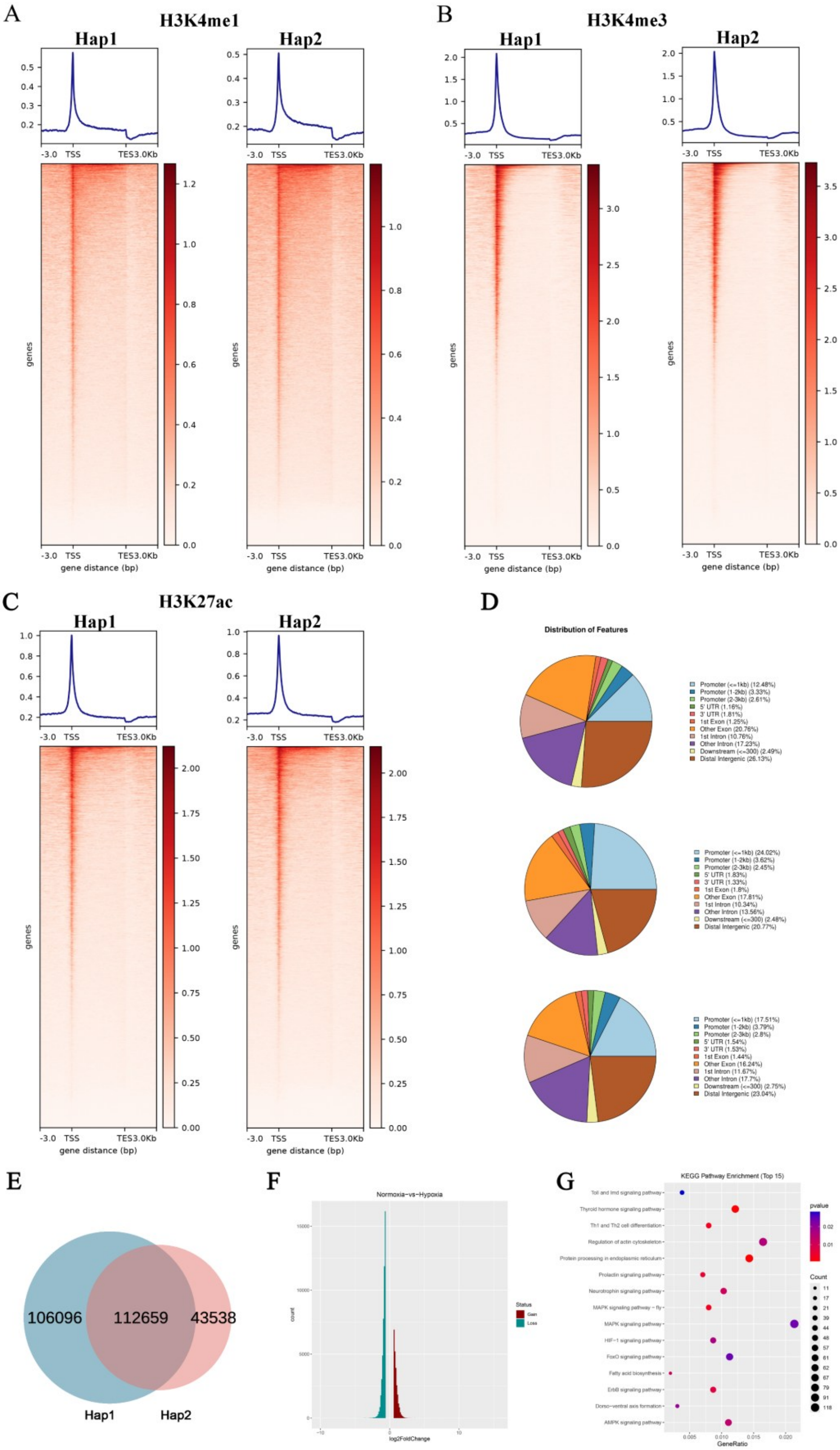

Fig. S17. Characteristics of CUT&Tag and ATAC sequencing signals in liver of *Larimichthys crocea.* (A) (B) (C) Profile plot and heatmap of CUT&Tag of H3K4me1,

H3K4me3, and H3K27ac signals near the Gene body region. (D) Distribution of CUT&Tag signals in different genomic regions. (E) Overlapping peak results of ATAC_seq of Hap1 and Hap2. (F) Distribution diagram of difference multiple and number of differentially accessible regions (DAR) of ATAC_seq of Hap1 and Hap2. (G) KEGG enrichment analysis of DAR-related genes.

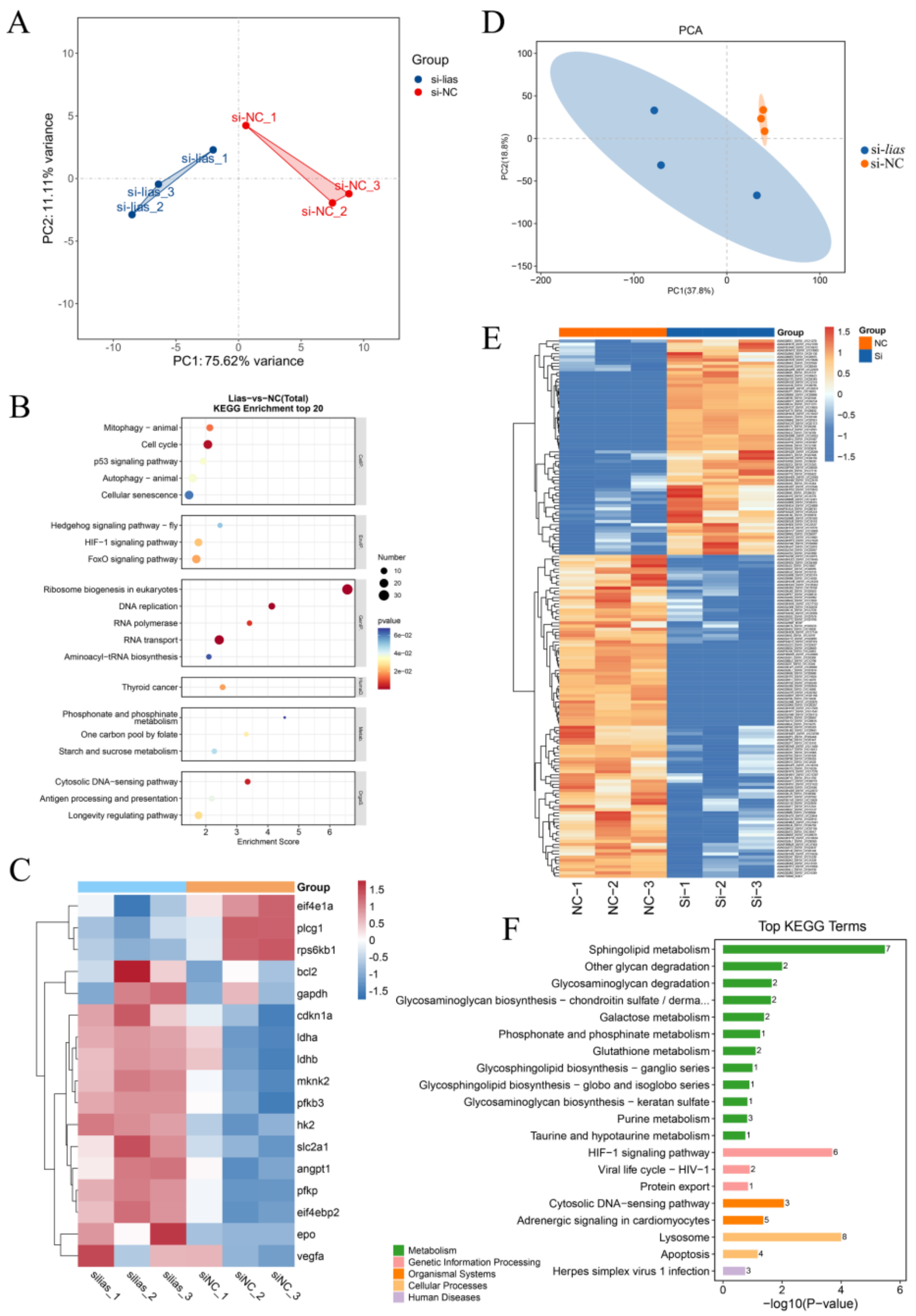

Fig. S18. Transcriptome and proteome analysis of LYCF cells with si_*lias* and si_NC group under hypoxia stress. (A) PCA plot of RNA-sequencing samples with si_*lias* and si_NC group. (B) Top 20 significantly enriched Kyoto Encyclopedia of Genes and Genomes (KEGG) pathways for DEGs. (C) Heatmap of DEGs in the HIF-1 signaling pathway. The heatmap shows statistically significant differences in gene expression profiles between si_*lias* and si_NC group. (D) PCA plot of proteomics samples with si_*lias* and si_NC group. (E) Heat maps of differentially abundant proteins (DAPs) between si_*lias* and si_NC group. (F) Top 20 significantly enriched KEGG pathways for DAPs.

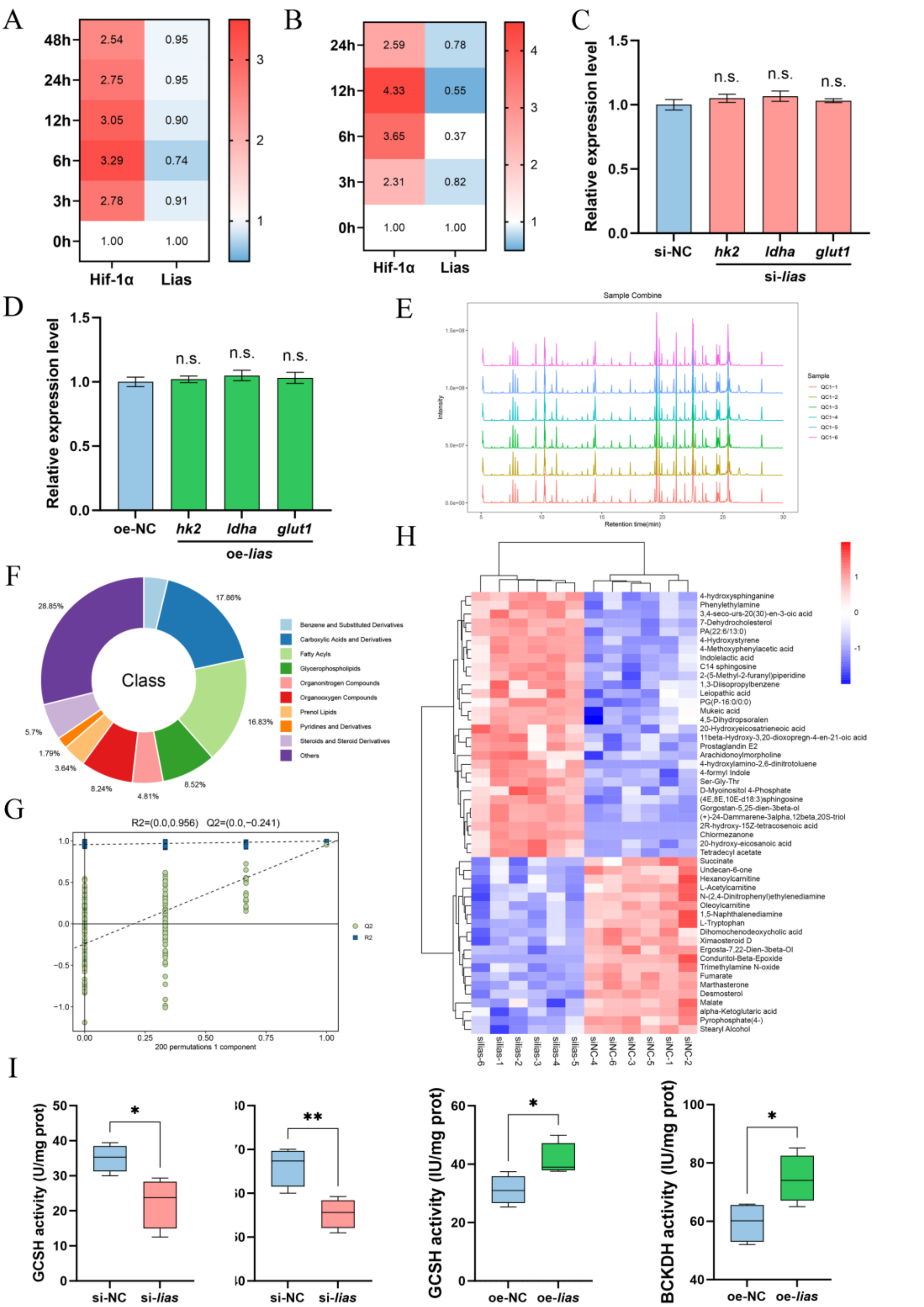

Fig. S19. Lias influences Hif-1α through metabolic pathways under hypoxic conditions. (A) (B) The heatmap displays the expression levels of Lias and Hif-1α proteins in liver tissue and LYCF cells of *Larimichthys crocea* under hypoxic stress conditions. (C) (D) The expression of Hif-1α downstream target genes (*hk2*, *ldha*, and *glut1*) in si-NC, si-*lias*, oe-NC, and oe-*lias* groups following Hif-1α interference under

hypoxic conditions. *hk2*, hexokinase 2; *ldha*, lactate dehydrogenase A chain; *glut1*, glucose transporter type 1. (E) Total ion current chromatogram (TIC) overlay of QC samples and classified pie charts of detected metabolites in GC-MS/LC-MS. (F) Pie plot of detected metabolites of metabolomics. (G) The plot of the replacement test results with metabolomics. The horizontal coordinates indicate the replacement retention, the vertical coordinates indicate the values of R2Y or Q2, the green dots indicate the R2Y values, the blue dots indicate the Q2 values, and the dashed line indicates the regression line. (H) Heat map of top 50 differential metabolite levels in GC-MS/LC-MS. (I) Changes of GCSH and BCKDH activities in si-NC, si-*lias*, oe-NC, and oe-*lias* groups under hypoxia stress. GCSH, glycine cleavage system H protein. BCKDH, branched-chain ketoacid dehydrogenase.

Table S14. Motif analysis of the CG2 region of the *lias* gene.

Rank	Motif	*P*_value	Name
1	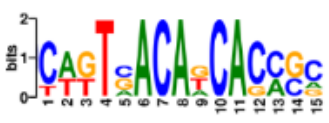	1.8E-12	MAFK
2	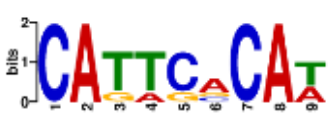	1.4e-05	DNTTIP1
3	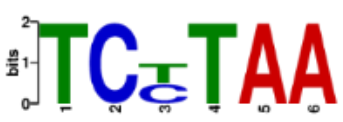	6.1e-04	ZNF827
4	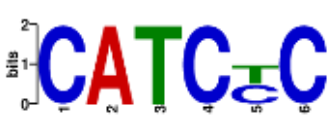	2.1e-02	ZNF410
5	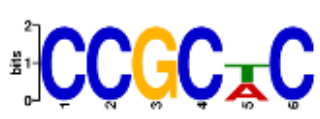	1.2e-01	ZNF610
6	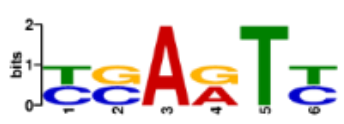	4.8e+01	NR1I2
